# Biological Degradation of Phthalates: From Bioremediation to Plastic Waste Valorization

**DOI:** 10.1002/cssc.202501955

**Published:** 2026-03-31

**Authors:** Marco A. Pereyra‐Camacho, Isabel Pardo

**Affiliations:** ^1^ Centro de Investigaciones Biológicas Margarita Salas CSIC Madrid Spain; ^2^ Interdisciplinary Platform SusPlast CSIC Madrid Spain

**Keywords:** biodegradation, bacteria, plasticizers, plastics, phthalates

## Abstract

Terephthalic, *ortho*‐phthalic, and isophthalic acid (i.e., phthalates) are aromatic petrochemicals with an important role in the plastics industry, since they are precursors for both polymers and plasticizers. Although their large‐scale industrial production is less than 80 years old, the recalcitrance and accumulation of plastics and plasticizers in the environment have caused severe pollution and health concerns. In this context, the biodegradation of these xenobiotic compounds and derived products has gained significant attention in recent years, as it could provide environmentally‐friendly solutions for the removal of these contaminants from the environment and for the biological recycling and valorization of phthalate‐derived plastic waste. Here, we review the known microbial mechanisms for the degradation of the three phthalate isomers and derived esters (polymers and plasticizers), focusing on bacterial hydrolases and catabolic pathways for the assimilation of the aromatic monomers. We then highlight recent advances made in the bioremediation of phthalates in the environment, the engineering of more efficient enzymes for the recycling of polymers and the degradation of plasticizers, the development of microbial cell factories for the valorization of phthalates, and the application of new biosensor tools. In all, this review highlights the potential of microbial biotechnology in achieving a circular economy for plastics.

## Graphical abstract

Phthalic acid isomers play a key role in the plastics industry, but pose significant environmental and health concerns. This review explores the microbial biodegradation of phthalates and their esters, focusing on bacterial enzymes, catabolic pathways, and cellular uptake mechanisms of phthalates. It highlights advances in native and engineered microorganisms for the bioremediation and upcycling of phthalates, and outlines future challenges toward sustainable plastics circularity.

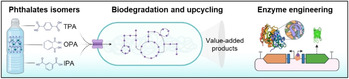

## 1 Introduction

**FIGURE 1:**
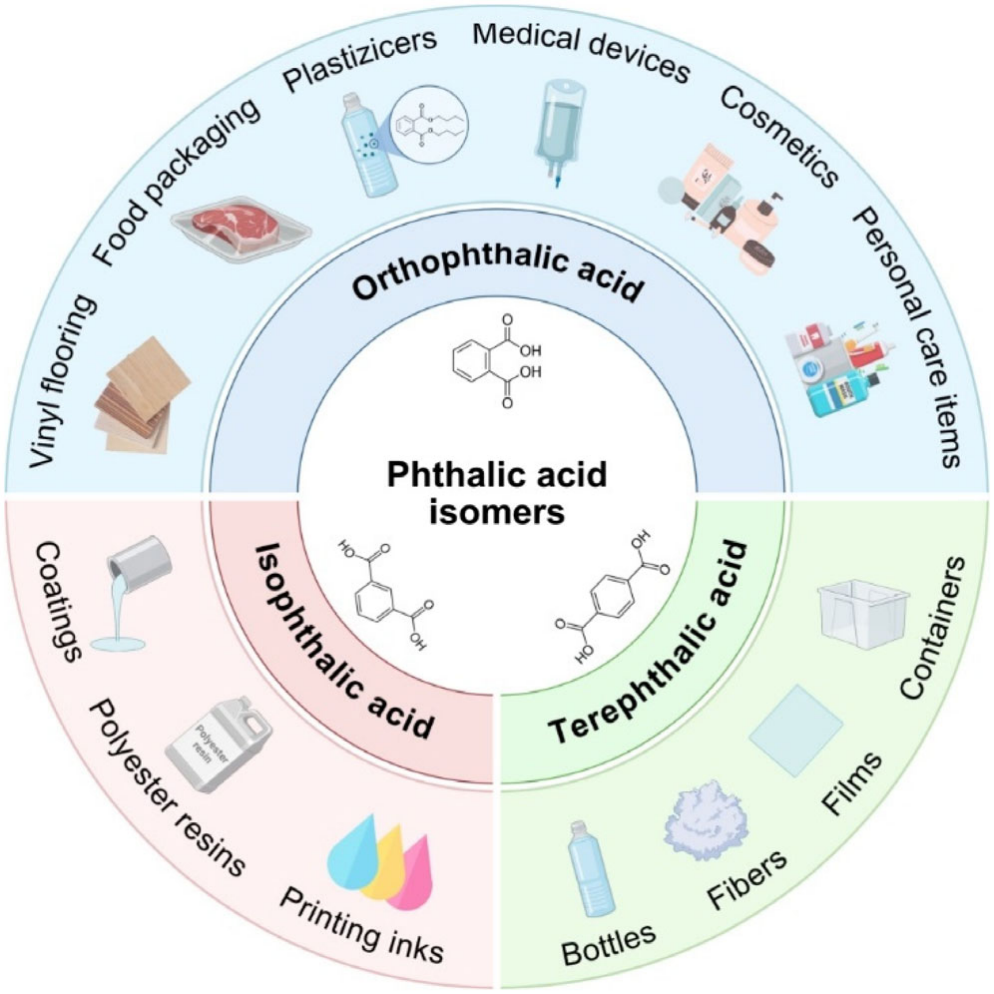
Industrial applications of phthalic acid isomers.

The three isomers of benzene dicarboxylic acid (i.e., *ortho*‐phthalic acid, *meta‐* or isophthalic acid, and *para‐* or terephthalic acid) are essential intermediates in the production of a broad range of plastic products, serving as precursors for both polymers and plasticizers. In particular, terephthalic acid (TPA) is most commonly used for the synthesis of polyethylene terephthalate (PET), a synthetic polyester with applications in textiles, beverage bottles, and food packaging. Isophthalic acid (IPA) is also used for polymer synthesis, mainly as co‐monomer in PET to improve its malleability and transparency. Lastly, *ortho*‐phthalic acid (OPA) is found in compounds generally known as “phthalic acid esters” (PAEs), which are diesters of OPA and are the most widely used plasticizers (Figure 1).

We should clarify that, although “phthalic acid” is commonly used in reference to OPA, and OPA‐based plasticizers are colloquially called “phthalates”, in this review we will use the general term “phthalates” to refer to all three benzene dicarboxylic acid isomers (i.e., OPA, IPA, and TPA), and refer to OPA‐based plasticizers as PAEs. Thus, phthalate‐derived products are relevant in numerous sectors, including packaging, construction, textiles, medical care, electronics, etc [1, 2, 3]. Due to their extensive applications, the global annual consumption of phthalates is estimated at approximately 90 million metric tons (Mt) for TPA, 5.6 Mt for OPA, and 1.3 Mt for IPA [4, 5, 6]. Despite recent regulation aimed at increasing the share of recycled plastic and limiting the production of single‐use plastics, as well as restrictions to the use of certain PAEs of concern for sensitive applications, it is expected that the demand for phthalates and phthalate‐derived products will continue to grow in the following years, in accordance with the trends of increasing plastics consumption in general [7].

Considering their relevance in the production of plastics, phthalate‐derived products play an important role in plastic pollution and human health. On one hand, PET is a recalcitrant polymer that can persist in the environment for decades or centuries, breaking down to micro‐ and nano‐plastics by slow fragmentation and erosion [8]. On the other hand, certain PAEs are considered substances of concern due to their endocrine disruption properties. Furthermore, as they are non‐covalently bound to plastic matrices, PAEs can leach into aquatic and terrestrial environments, causing negative effects on both wildlife and humans [9, 10, 11].

Despite the anthropogenic origin of most phthalates and related compounds, and the relatively short period of time for which they have been accumulating in the environment, their degradation mediated by different microorganisms has been known for several decades. Notably, some of the earlier reports on the biodegradation of phthalates date back to the 1970s, when awareness on the accumulation of PAEs in the environment and the potential risk to human health began to emerge [12]. Particularly in the case of bacteria, enzymes mediating the hydrolysis of phthalate polyesters and diesters, as well as catabolic pathways for their assimilation, have been identified and described in more or less detail depending on the phthalate isomer. These findings have opened the door to innovative approaches based on the use of bacteria for the bioremediation of phthalates and the biological recycling and upcycling of plastic waste.

Here, we will briefly introduce the relevance of phthalates in the plastics industry and their impact on the environment and human health. We will then deep‐dive into the current knowledge on the microbial biodegradation of phthalates and phthalate‐derived products—namely polyesters and plasticizers—with a special focus on bacterial hydrolytic enzymes and catabolic pathways required for the assimilation of the three phthalate isomers. We will also address recent advances made in the use of native and engineered microorganisms and their enzymes to eliminate phthalate‐related products from the environment and to improve the circularity of the plastic industry, both by enzymatic recycling and microbial upcycling of plastic waste to value‐added bioproducts. Lastly, we will discuss future research directions and identify the limitations and knowledge gaps that will need to be overcome to fully leverage the potential of microorganisms for the bioremediation and biological valorization of phthalate‐derived products.

### 1.1 Applications of Phthalates

Phthalates are petrochemicals whose large‐scale industrial synthesis mostly dates back to the second half of the 20th century, although commercial production of phthalate anhydride (the main precursor form in which OPA is produced) was already implemented by BASF in 1872 using naphthalene as feedstock. Today, most TPA, IPA, and phthalic anhydride are synthesized via oxidation of *para‐*, *meta*‐, and *ortho*‐xylene, respectively [1, 2]. These aromatic compounds are largely used as precursors for polyesters or diesters, finding their main application in the plastic and textile industries (Figure 1). Most TPA is used in the synthesis of the polyester PET for the manufacture of fibers, beverage bottles, and food packaging. Approximately two‐thirds of PET is used in textiles, where PET is usually referred to as simply polyester. Indeed, polyester is the most widely produced synthetic fiber globally, accounting for 57% of the market share in 2023 [13]. Other relevant polymers produced from TPA include polybutylene terephthalate (PBT), a high‐performance molding resin, and polybutylene adipate terephthalate (PBAT), a biodegradable plastic largely used in agricultural mulch films. Some TPA diesters are also used as plasticizer alternatives to low‐molecular‐weight PAEs. Similarly, most IPA is also used as a co‐monomer for the production of PET bottles, as it decreases polymer crystallinity and provides increased transparency and malleability. To a lesser extent, it is used in coatings and high‐performance unsaturated polyesters [2].

As for OPA, it is the common moiety in PAEs, which are the plasticizers used in 70% of the global market [3]. These compounds play a crucial role in enhancing the flexibility and durability of plastic materials, most prominently polyvinyl chloride (PVC). Additionally, they are also used as plasticizers for coatings, epoxy resins, cosmetics, and personal care products. Thus, PAEs are largely employed in a wide range of consumer goods, from floor tiles and medical devices to food containers and children's toys. However, some of the historically most used PAEs are now considered substances of very high concern (see Section 1.2). Growing regulation on the use of these PAEs for sensitive applications such as toys, medical, and food contact applications has led to the search for alternative plasticizers, including high‐molecular weight PAEs or TPA and adipate esters, to name a few. However, the performance of these alternative plasticizers is somewhat lower than that of PAEs, requiring higher loadings and limiting their applicability [3].

### 1.2 Environmental and Health Concerns Caused by Phthalates

The negative effects of phthalate‐derived products are mainly caused by the accumulation of recalcitrant PET and the leaching of toxic PAEs in the environment. Similar to other conventional plastics, PET is extremely resistant to degradation (especially materials with crystallinity > 30%). Since the rates at which plastics are emitted into the environment far exceed those of removal, this leads to an accumulation of plastic pollutants that is poorly reversible [14]. In general, it is considered that abiotic weathering and photodegradation are the main mechanisms by which plastics are degraded in the environment, breaking down into fragments of smaller size that can be ingested by animals or colonized by different microorganisms. In particular, the ingestion of plastics by marine macrofauna has been extensively documented, including species at risk of extinction [14, 15]. Through the combined action of these abiotic and biotic processes, weathered plastics—including PET—break down into microfibers (MFs) and micro‐ and nanoplastics (MNPs), and their physical, chemical, and mechanical properties are changed [16, 17]. MFs and MNPs can be easily dispersed, eventually entering the food chain and accumulating in the bodies of larger animals. Indeed, MFs and MNPs have been found in almost all evaluated environments, including the human body. However, there is still no consensus within the scientific community as to whether MFs and MNPs are hazardous to human health in themselves, other than the possible release of toxic additives and their ability to act as vectors for harmful chemicals and pathogens [18].

**FIGURE 2:**
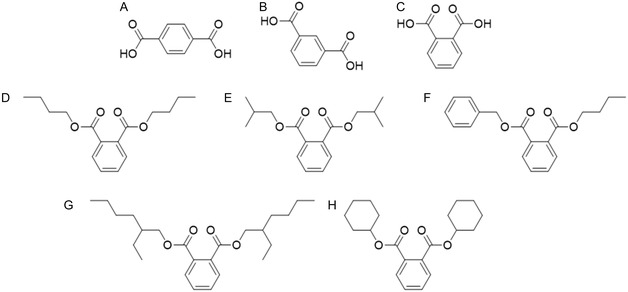
Chemical structures of the three phthalate isomers and PAEs classified as endocrine disrupting substances by the European Chemicals Agency (ECHA). (A) Terephthalic acid (TPA). (B) Isophthalic acid (IPA). (C) Orthophthalic acid (OPA). (D) Dibutyl phthalate (DBP). (E) Diisobutyl phthalate (DIBP). (F) Benzyl butyl phthalate (BBP). (G) Di(2‐ethylhexyl) phthalate (DEHP). (H) Dicyclohexyl phthalate (DCHP).

On the other hand, the main concern caused by PAEs is that they can be toxic to reproduction, although the mechanisms behind their endocrine‐disrupting properties are still unclear [1]. While PAE exposure has been linked to numerous health risks in humans, including cardiovascular and respiratory diseases, obesity, and diabetes, epidemiological studies only demonstrate consistent association with decreased sperm quality, leading to male infertility [9]. This effect is proposed to be caused by their interference with the normal functioning of the hypothalamic–pituitary‐gonadal hormonal axis. Notably, most studies have focused on di(2‐ethylhexyl) phthalate (DEHP), the most historically used plasticizer [19]. In the case of this PAE, toxicity seems to be mainly exerted by mono(2‐ethylhexyl) phthalate, as one of the alkyl chains of DEHP is rapidly hydrolyzed and the resulting monoester is typically detected in higher levels in biological samples [1, 9]. As they are not covalently bound to the plastic materials, PAEs can leach into the surrounding environment. Routes of human exposure include oral ingestion, inhalation, and dermal contact. The European Union (EU), the US Environmental Protection Agency (USEPA), the China National Environmental Monitoring Center, and various international organizations have classified several PAEs as priority environmental pollutants. Particularly in the EU, 14 PAEs are included within Annex XIV of the REACH Authorization List, meaning that manufacturers must request a specific authorization to continue using these controlled substances before their use is prohibited [20]. Of these, five PAEs are included in the Candidate List of substances of very high concern due to their endocrine‐disrupting properties: benzyl butyl phthalate (BBP), bis(2‐ethylhexyl) phthalate (DEHP), dibutyl phthalate (DBP), diisobutyl phthalate (DIBP), and dicyclohexyl phthalate (DCHP) (Figure 2). Nevertheless, it is likely that more PAEs and PAE‐like substances will be regulated in the near future as more evidence on their hazardous effects becomes available, stressing the need for safer alternatives for these widely used plasticizers.

## 2 Mechanisms for the Microbial Degradation of Phthalate‐Related Products

Thanks to the presence of hydrolyzable bonds, phthalate polyesters and diesters are susceptible to biodegradation in the environment, although this process usually proceeds slowly depending on external factors such as temperature, light exposure, water and oxygen availability, etc. In the case of PET, it is estimated that its half‐life can span from 2.3 years to >2500 years, depending on the environment and the exposure to UV light and heat [8]. In contrast, PBAT is fully compostable under industrial conditions according to international standards, and can be biodegradable in home compost and soil depending on the formulation [21]. The main limitation to the biodegradation of PET lies in its hydrophobicity and crystallinity, which hinder biofilm formation on the material surface and the access of secreted enzymes to the polymer chains [22, 23].

Compared to PET, PAEs are degraded much more rapidly, and biodegradation is the main fate of these compounds when they are released in the environment over abiotic processes such as photooxidation or hydrolysis [24, 25]. Additionally, the lower molecular mass of PAEs allows them to translocate through cell membranes by passive diffusion or through specialized transporters [26, 27]. Thus, hydrolysis can proceed within the cell, and the released alcohols and OPA can be assimilated through dedicated catabolic routes. Of note, several low‐molecular‐weight PAEs have been found to be naturally synthesized as bioactive compounds by higher plants, algae, fungi, and bacteria [28]. Thus, it is possible that the microbial catabolism of the different phthalate isomers has evolved from degradation mechanisms for the detoxification of these and other structurally‐related compounds.

In any case, the common steps in the microbial degradation of phthalate‐derived poly‐ and diesters are i) the hydrolysis of ester bonds, releasing the corresponding phthalate and alcohols, and ii) the assimilation of the released monomers as carbon and energy sources for the cell. In this section, we review the current knowledge regarding these two steps.

### 2.1 Enzymatic Hydrolysis of Phthalate‐Ester Bonds

The degradation of phthalate polyesters and diesters begins with their hydrolysis, a process typically catalyzed by different members of the alpha/beta hydrolase family. Here, we will separately consider enzymes acting on polymers (namely, PET hydrolases) and PAE hydrolases. Over the last decade, there have been enormous advances in the field with regards to the discovery, characterization, and engineering of PET hydrolases, and these have been extensively reviewed elsewhere [29, 30]. In contrast, studies on PAE hydrolases are much scarcer and, to our knowledge, there are no reports on enzymes that specifically hydrolyze IPA esters. Thus, here we will describe PAE hydrolases in more detail.

#### 2.1.1 PET Hydrolases

The first reports on the enzymatic degradation of PET date back to 2005, when a hydrolase from *Thermobifida fusca* was shown to mediate the weight loss of PET films [31]. Since then, the number of enzymes reported to depolymerize PET to the constituent monomers TPA and ethylene glycol (EG) has increased exponentially. A major gamechanger was the isolation of a bacterium capable of depolymerizing and mineralizing PET at ambient temperature [32]. This milestone opened the door to a flood of publications describing the engineering of hydrolases for the enzymatic recycling of PET, even though this research had steadily continued up to the identification of *Ideonella sakaiensis*’ PETase in 2016 (*Is*PETase, now reclassified as *Piscinibacter sakaiensis*) (see Section 3.2). Beyond *Is*PETase, the most well studied PET hydrolases include thermophilic cutinases such as the leaf‐branch compost cutinase (LCC), isolated from a metagenomic library; or Cut190 from *Sacharomonospora viridis* [33, 34].

It is generally assumed that the most efficient PET hydrolases belong to the cutinase family, and that the ability to hydrolyze PET is the result of non‐specific activity [35]. Cutin is an aliphatic polyester mainly composed of C16‐C18 ω‐hydroxy fatty acids that is found in the plant cuticle, acting as a hydrophobic layer that coats the outer surface of the plant's aerial organs [36]. The main difference between conventional cutinases and what are considered PET hydrolases is presumed to lie in their ability to accommodate the bulkier aromatic TPA moiety in PET. Since the PET polymer is too large to be internalized by the cell, PET hydrolases are generally secreted. Enzymes that are active on PET have been identified from many different environments, highlighting the enormous hydrolytic potential of microorganisms for the biodegradation of synthetic polymers. To date, the Plastics‐Active Enzymes Database (PAZy) collects 124 known and biochemically characterized wild‐type PET hydrolases, the majority of which are of bacterial origin [37].

However, the main limitation to the enzymatic degradation of PET is its crystallinity. Despite the fact that some psychrophilic and mesophilic enzymes have been shown to degrade PET to some extent at moderate temperatures, efficient depolymerization of this synthetic polymer generally requires reaction temperatures close to the polymer's melting temperature (˜70°C) and a pre‐treatment to amorphosize PET (<15% crystallinity). Depolymerization yields decrease dramatically as polymer crystallinity increases, and the crystallinity of commercial PET bottles and fibers is typically above 30% [38, 39]. Thus, it follows that the majority of PET in the environment will be impervious to enzymatic attack [22]. Nevertheless, numerous efforts have been dedicated to engineering more efficient PET hydrolases, with the goal of developing either enzymatic recycling processes or bioremediation strategies to reduce PET pollution in the environment (see Section 3.2).

**FIGURE 3:**
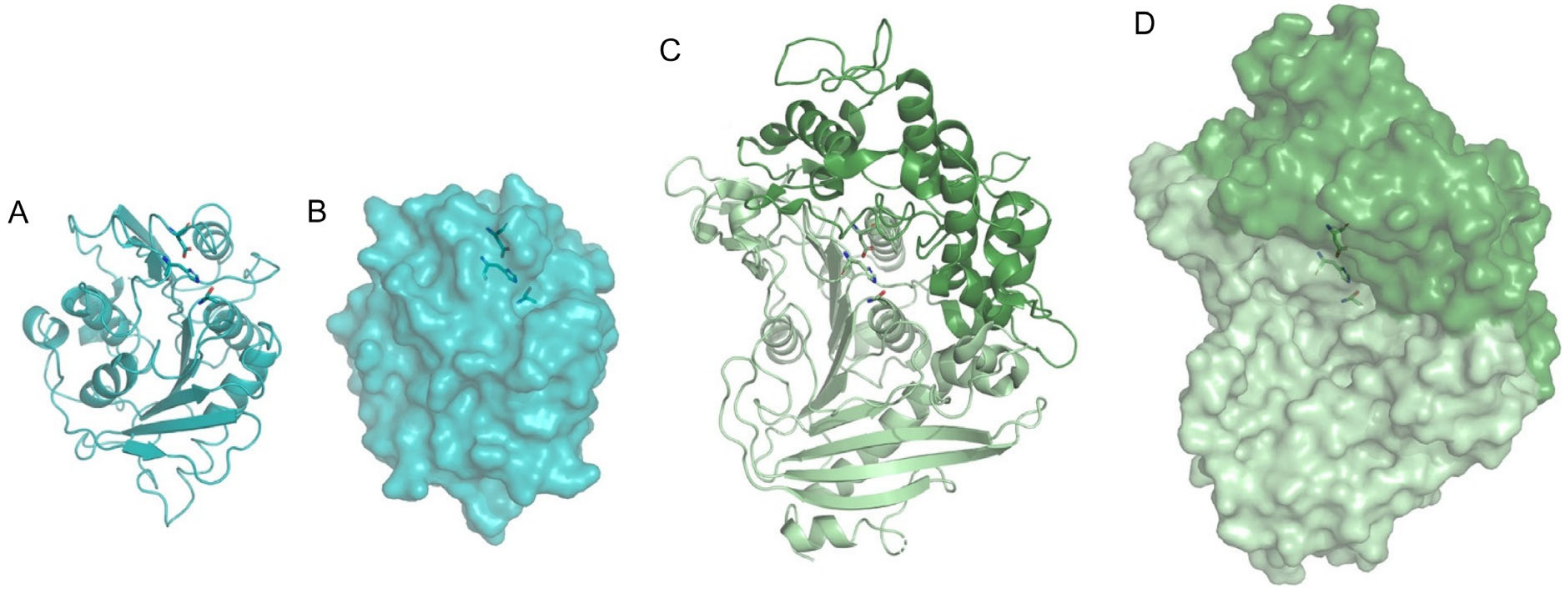
(A,B) Tridimensional structures of PETase (PDB 6EQE) and (C,D) MHETase (PDB 6QGA) from *I. sakaiensis*. The lid domain of MHETase is shown in dark green. Catalytic triads formed by Ser‐Asp‐His residues are shown as sticks.

The enzymatic hydrolysis of PET is generally considered to proceed via random endo‐chain scission. Enzymatic attack is initiated on the more easily accessible polymer chains of the surface, releasing oligomers of lower molecular mass and increased solubility, which can be hydrolyzed more efficiently [40]. Typically, an accumulation of mono(2‐hydroxyethyl) terephthalate (MHET) is observed. Recent studies have shown that the enzymatic hydrolysis of MHET by PET hydrolases can be orders of magnitude less efficient than the hydrolysis of oligomers with at least 3 TPA units [39]. Indeed, *I. sakaiensis* encodes a MHETase enzyme related to feruloyl esterases that specifically hydrolyzes MHET to TPA and EG, and some works have shown that complete PET depolymerization is enhanced by the synergistic action of PETase and MHETase [41, 42]. In contrast to PETase, which shows a shallow, solvent exposed cleft that allows it to bind the PET polymer chain, MHETase possesses a lid domain to specifically and efficiently position MHET for hydrolysis (Figure 3) [43]. Of note, a recent work has also described the identification of BHETases, which preferentially attack bis‐2‐hydroxyethyl terephthalate (BHET). These enzymes have been further engineered for improved activity and thermostability, demonstrating improved free TPA yields when used in combination with different PETases [44].

#### 2.1.2 PAE Hydrolases

Unlike PET, PAEs can be small enough to be incorporated by microbial cells through unspecific or specific mechanisms (see Section 2.3). Accordingly, PAE hydrolases identified to date are either known or predicted to be intracellular, due to the lack of conserved secretion signal peptides. The enzymatic degradation of PAEs involves two hydrolytic steps, sequentially catalyzed by a diesterase and a monoesterase or by diesterase‐monoesterases capable of carrying out both reactions. However, for the latter, the monoesterase reaction is generally less efficient than the diesterase reaction [45, 46], as described above for the PETase‐MHETase system from *I. sakaiensis*.

**FIGURE 4:**
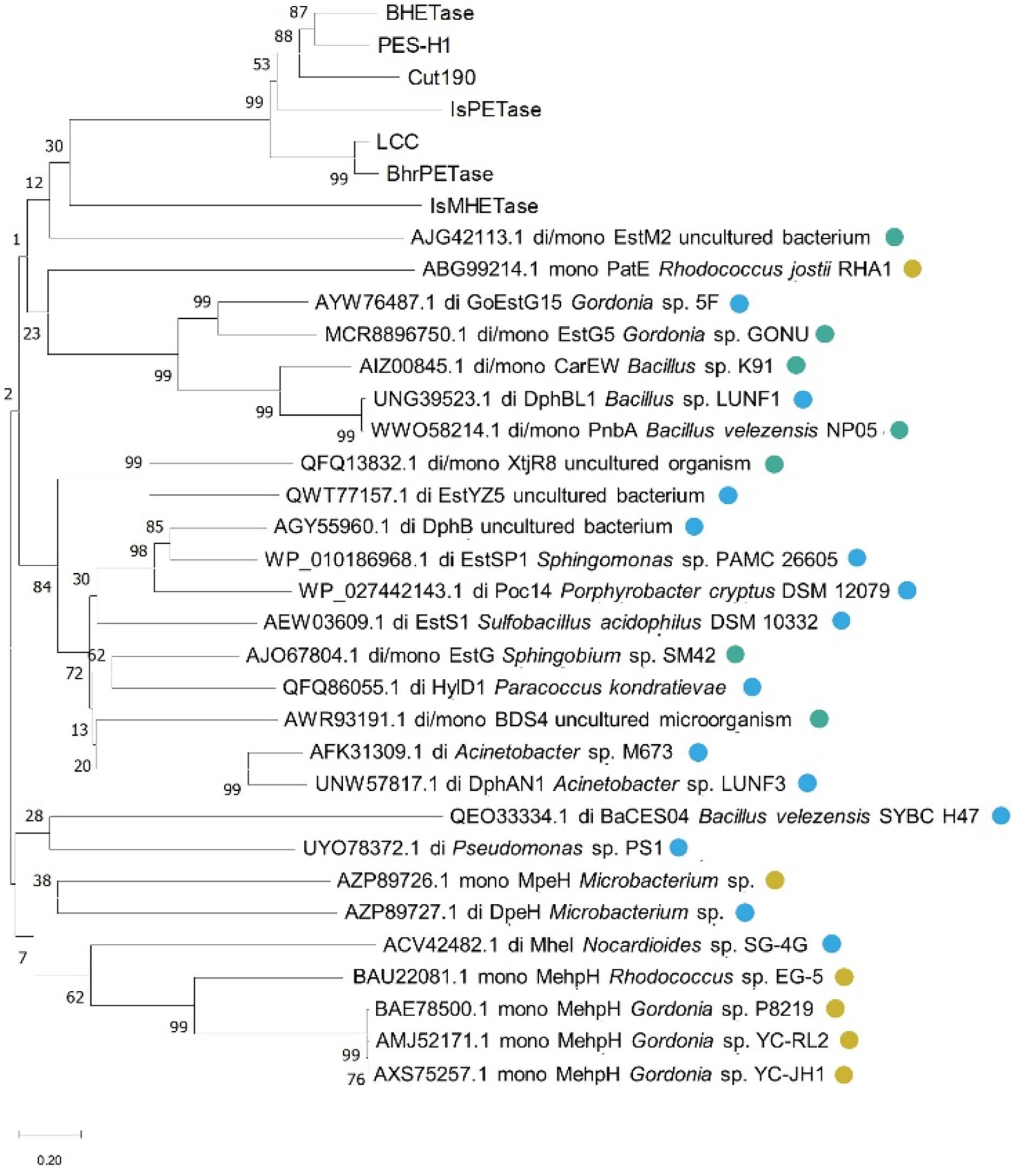
Phylogenetic analysis of experimentally validated PAE hydrolases. Enzymes described as monoesterases (mono), diesterases (di), or diestereases/monoesterases (di/mono) are indicated with yellow, blue, and green circles, respectively. PET, BHET, and MHET hydrolases mentioned in the main text are also included in the analysis. The evolutionary analysis was performed in MEGA11 using the Neighbor Joining method with a bootstrap of 500 iterations [72]. The numbers at the nodes indicate the percentage of trees in which the associated taxa clustered together. Branch lengths are scaled to the number of substitutions per site.

To date, there are 27 bacterial PAE hydrolases that have been experimentally validated and whose sequences are deposited in databases (Table 1). Phylogenetic analysis of their sequences reveals two main clusters that differentiate enzymes reported to act as monoesterases from those described as diesterases and diesterases‐monoesterases, with few exceptions (Figure 4). However, most PAE hydrolases have not been characterized in detail, and to our knowledge, no systematic comparison of substrate specificity and reaction rates have been performed for these enzymes. This knowledge gap will need to be addressed in order to identify the most efficient enzymes for the hydrolysis of PAE and develop biocatalytic processes to degrade these pervasive pollutants, much in the same way as has been done in recent years for PET hydrolases.

**TABLE 1:** Biochemically characterized PAE hydrolases with deposited sequences in the National Center for Biotechnology Information (NCBI).

Activity	Enzyme name	Substrates	Source	ESTHER classification	Accession no.	Reference
Monoesterase	MehpH	MEHP, MHP, MBP, MEP	*Gordonia* sp. P8219	Carbon‐carbon_bond_hydrolase	BAE78500	[47]
	PatE	MMP, MBP, MHP, MEHP	*Rhodococcus jostii* RHA1	N.A.	ABG99214	[48]
	MehpH	MEHP, MHP, MBP, MEP	*Rhodococcus* sp. EG‐5	Carbon‐carbon_bond_hydrolase	BAU22081	[49]
	MehpH	MEHP, MBP, MMP, MEP	*Gordonia* sp. YC‐RL2	Carbon‐carbon_bond_hydrolase	AMJ52171	[50]
	MphG1	MEP, MBP, MHP, MEHP	*Gordonia* sp. YC‐JH1	Carbon‐carbon_bond_hydrolase	AXS75257	[51]
	MpeH	MMP, MEP, MBP, MPP, MBzP, MHP, MEHP	*Microbacterium* sp. PAE‐1	Bacterial_esterase	AZP89726	[52]
Diesterase	DphB	DPrP, DBP, DPP	Metagenome	Hormone‐sensitive_lipase_like	AGY55960	[53]
	N.A.	DMP, DEP, DPrP, DBP, DPP, DHP	*Acinetobacter* sp. M673	Hormone‐sensitive_lipase_like	AFK31309	[54]
	EstS1	DEP, DPrP, DBP, DPeP, DHP, BBP, DEHP	*Sulfobacillus acidophilus*	Hormone‐sensitive_lipase_like	AEW03609	[55, 56]
	EstSP1	DEP, DBP, DHP	*Sphingomonas* sp. PAMC 26 605	Hormone‐sensitive_lipase_like	WP_010186968	[57]
	GoEst15	DMP, DEP, DPrP, DBP, DiBP, BBP, DPP, DHXP, DNOP, DEHP, DCHP	*Gordonia* sp. 5F	Carb_B_Bacteria	AYW76487	[58]
	BaCEs04	DMP, DEP, DPrP, DBP	*Bacillus velezensis* SYBC H47	A85‐IroE‐IroD‐Fes‐Yiel	QEO33334	[59]
	DpeH	DMP, DEP, DBP, DPP, BBP, DHP, DEHP	*Microbacterium* sp. PAE‐1	HNLyase_Bact	AZP89727	[52]
	HylD1	DMP, DEP	*Paracoccus kondratievae*	Hormone‐sensitive_lipase_like	QFQ86055	[60]
	EstYZ5	DMP, DEP, DBP, DAP, BBP, DEHP	Metagenome	Hormone‐sensitive_lipase_like	QWT77157	[61]
	PS06828	DBP, DEHP	*Pseudomonas* sp. PS1	LYsophospholipase_carboxylesterase	UYO78372	[62]
	DphBL1	DBP, BBP, DEP	*Bacillus* sp. LUNF1	Carb_B_Bacteria	UNG39523	[63]
	DphAN1	DEP, DBP, BBP	*Acinetobacter* sp. LUNF3	Hormone‐sensitive_lipase_like	UNW57817	[64]
	MheI	DMP	*Nocarsioides* sp. SG‐4G	HNLyase_Bact	ACV42482	[65]
	Poc14	DEP	*Porphyrobacter cryptus* DSM12079	Hormone‐sensitive_lipase_like	WP_027442143	[66]
Monoesterase/ Diesterase	CarEW	DiBP, MiBP	*Bacillus* sp. K91	Carb_B_Bacteria	AIZ00845	[67]
	EstG	DBP, MBP	*Sphingobium* sp. SM42	Hormone‐sensitive_lipase_like	AJO67804	[45]
	BDS4	DMP, DEP, DBP	Metagenome	Hormone‐sensitive_lipase_like	AWR93191	[68]
	EstJ6	DMP, DEP, DPrP, DBP, DPP, DHP, MMP, MEP, MBP, MHP	Metagenome	Hormone‐sensitive_lipase_like	QCQ29100	[69]
	XtjR8	DBP, DPP, DEP, DPrP, DMP, DHP, MBP	Metagenome	Hormone‐sensitive_lipase_like	QFQ13832	[46]
	EstM2	DBP, BBP, DPP, DEP, DMP, MBP, MBzP, MPP, MEP, MMP	Metagenome	N.A.	AJG42113	[70]
	PnbA	DiBP	*Bacillus velezensis* NP05	Carb_B_Bacteria	WWO58214	[71]

Abbreviations: MEHP: mono(2‐ethylhexyl) phthalate; MHP: mono‐n‐hexyl phthalate; MBP: mono‐n‐butyl phthalate; MEP: mono‐n‐ethyl phthalate; MMP: mono‐n‐methyl phthalate; MPP: mono‐n‐pentyl phthalate; MBzP: mono‐n‐benzyl phthalate; MiBP: mono‐isobutyl phthalate; DEP: diethyl phthalate; DBP: dibutyl phthalate; BBP: benzyl butyl phthalate; DMP: dimethyl phthalate; DPrP: dipropyl phthalate; DPP: dipentyl phthalate; DHP: dihexyl phthalate; DIBP: diisobutyl phthalate; DHXP: di‐n‐hexyl phthalate; DNOP: di‐n‐octyl phthalate; DEHP: di(2‐ethylhexyl) phthalate; DCHP: dicyclohexyl phthalate; DAP: diallyl Phthalate; DPeP: di‐n‐pentyl phthalate; N.A. Not applicable.

**FIGURE 5:**
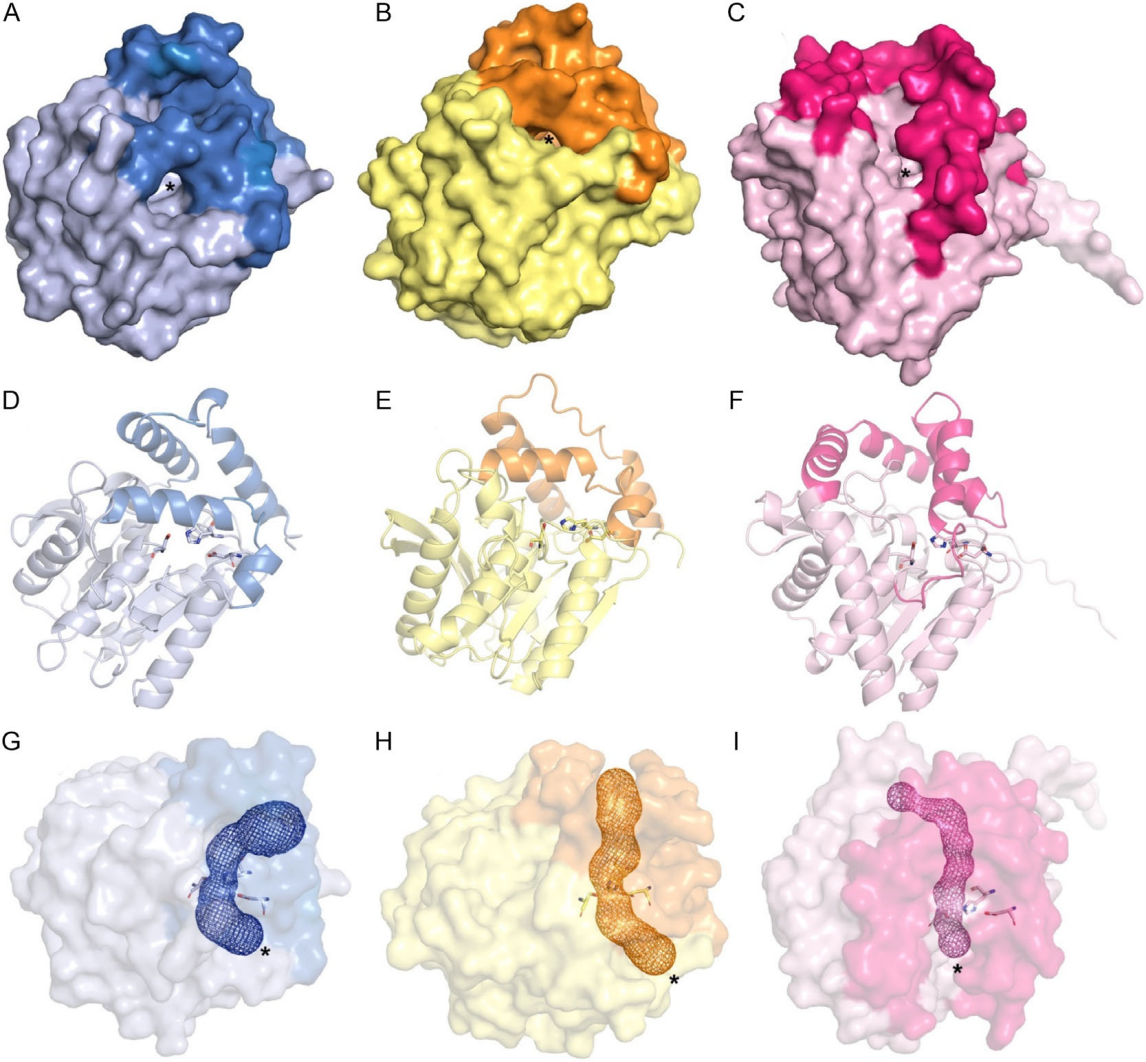
(A,D,G) Tridimensional structure models of HylD1 diesterease from *Paracoccus kondratievae*, (B,E,H) Est1 diesterase from *Sulfobacillus acidophilus*, and (C,F,I) MhepH1 monoesterase from *Gordonia* sp. P8219. Since the deposited crystal structures are missing some residues, AlphaFold models are shown instead. Helixes annotated as “cap” or “lid” domains are shown in darker colors. (A–C) Surface representation. Putative substrate entry pockets are marked with an asterisk. (D–E) Cartoon representation. Catalytic triads formed by Ser‐Asp‐His residues are shown as sticks. (G–I) Putative tunnels identified with Caver 3.0 shown in mesh representation. [73] Structures are rotated with respect to panels A–F for better visualization.

As inferred from the cladogram shown in Figure 4, PAE monoesterases and PAE diesteresases and diesterases/monoesterases seem to have a different evolutionary origin. According to the ESTHER database, most diesterases and diesterases/monoesterases are classified as hormone‐sensitive lipase‐like enzymes, whereas the majority of monoesterases are classified as carbon–carbon bond hydrolases (Table 1). This difference is also appreciated in their overall protein structures. Figure 5 shows the crystal structures of PAE hydrolases reported do date: the monoesterase MehpH from *Gordonia* sp. P8219 and the diesterases HylD1 and EstS1 from *Paracoccus kondratievae* and *Sulfobacillus acidophilus*, respectively [56, 74, 75]. We note that during the preparation of this manuscript, the crystal structure of another diesterase was also solved, namely Poc14 from *Porphyrobacter cryptus* DSM12079 [66]. However, its structure is not analyzed here. While these three enzymes present a conserved alpha‐beta hydrolase core, differences can be found in what is known as the “lid” or “cap” domain, which helps position the substrate in the catalytic site to facilitate its hydrolysis and is presumed to determine substrate specificity in this family of enzymes. Similarly, the potential tunnels for substrate entry and product egress are different between the monoesterase MehpH and the diesterases HylD1 and EstS1, which is seemingly narrower and straighter in the former. This difference could account for the distinct monoester or diester specificity, as the wider tunnel could potentially accommodate bulkier substrates with two alkyl chains. The steric hindrance imposed by these tunnels could also explain the differences in hydrolytic efficiency over low‐ and high‐molecular weight PAEs.

### 2.2 Assimilation Pathways of Phthalates

Through the course of evolution, bacteria have acquired the ability to assimilate a wide range of compounds. This is possible thanks to what is known as “catabolic funneling”. In the case of the catabolic pathways for aromatics, these can be distinguished between “upper” or “peripheral” pathways, which convert different compounds into a few common aromatic intermediates; and “lower” pathways, in which the aromatic ring is opened and converted to intermediates of central metabolism that are diverted towards the production of biomass and energy [76]. Conserved pathways for the biodegradation of the three phthalate isomers have been identified in aerobic and anaerobic bacteria, although the latter have not been as extensively studied or characterized in detail. In general, aerobic pathways are considered to be more efficient, as anaerobic pathways require metabolically expensive molecules such as coenzyme A and ATP to activate the aromatic ring and facilitate decarboxylation and dearomatization of the substrate [77].

### 2.3 Aerobic Degradation Pathways

**FIGURE 6:**
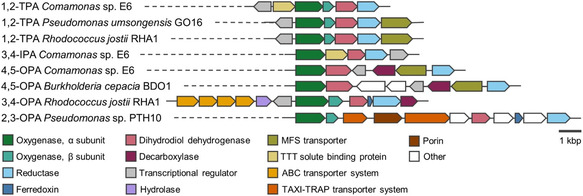
Schematic representation of the organization of representative genetic clusters for the degradation of phthalate isomers in aerobic bacteria.

**SCHEME 1:**
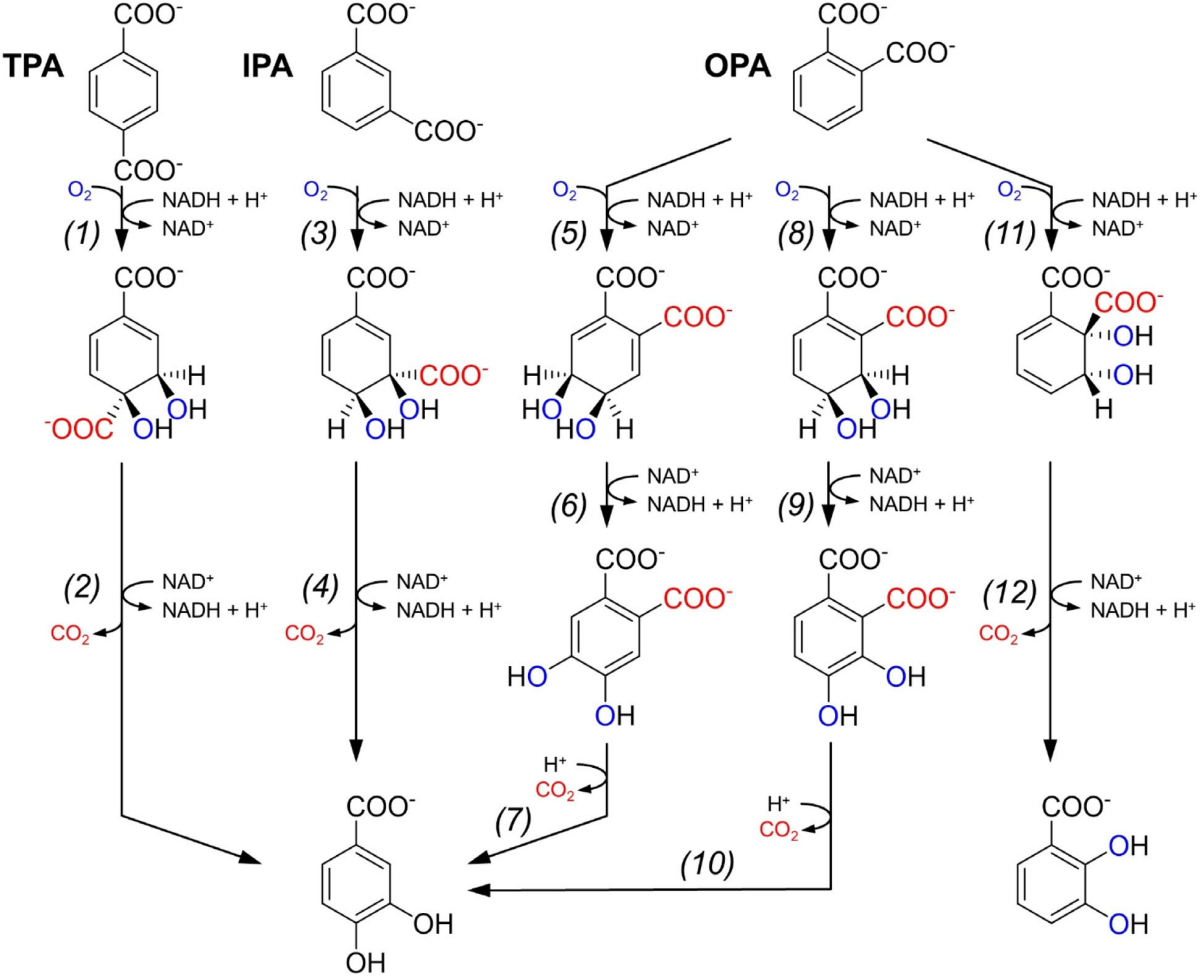
Catabolic pathways for the aerobic assimilation of phthalates in bacteria. From left to right, 1,2‐TPA dioxygenase pathway, 3,4‐IPA dioxygenase pathway, 4,5‐OPA dioxygenase pathway, 3,4‐OPA dioxygenase pathway, and 2,3‐OPA dioxygenase pathway. Numbers in parentheses indicate the following enzymes: (*1*) terephthalate 1,2‐dioxygenase; (*2*) *cis*−1,2‐dihydrodiolterephthalate dehydrogenase; (3) isophthalate 3,4‐dioxygenase; (*4*) *cis*−1,2‐dihydrodiolisophthalate dehydrogenase; (*5*) orthophthalate 4,5‐dioxygenase; (*6*) phthalate *cis*−4,5‐dihydrodiol dehydrogenase; (*7*) 4,5‐dihydroxyphthalate decarboxylase; (*8*) orthophthalate 3,4‐dioxygenase; (*9*) phthalate *cis*−3,4‐dihydrodiol dehydrogenase; (*10*) 3,4‐dihydroxyphthalate decarboxylase; (*11*) orthophthalate 2,3‐dioxygenase; (*12*) phthalate *cis*−2,3‐dihydrodiol dehydrogenase.

In most aerobic bacteria, all three phthalate isomers are converted to protocatechuic acid (PCA) through sequential reactions of dihydroxylation, dehydrogenation, and decarboxylation (Scheme 1). PCA is a common intermediate in the assimilation of several aromatic substrates in aerobic bacteria, and is the substrate for dioxygenase enzymes capable of cleaving the phenolic ring in different positions. Subsequent enzymatic steps allow converting the products of PCA ring‐cleavage into different intermediates of central metabolism [76, 78]. Phylogenetic analysis of the gene clusters involved in the different phthalate degradation pathways suggests distinct evolutionary origins, although in all cases the initial dihydroxylation is carried out by Rieske non‐heme iron oxygenases [79]. In the case of TPA and IPA, dehydrogenation and decarboxylation of the dihydrodiol intermediate to PCA occur in a single enzymatic step, whereas for OPA, two enzymatic steps are required. The enzymes responsible for the conversion of TPA, IPA, and OPA, respectively, are surprisingly conserved among aerobic bacteria, and they are encoded in genetic clusters that maintain a similar overall organization (Figure 6). Furthermore, they are often surrounded by mobile genetic elements such as transposases or integrases, suggesting their acquisition through horizontal gene transfer. However, some differences can be found regarding transport (see Section 2.3).

The first catabolic step in the degradation of TPA is dihydroxylation by a two‐component Rieske oxygenase system (terephthalate 1,2‐dioxygenase or TPADO), composed of large (α) and small (β) oxygenase subunits and their cognate reductase. This enzyme complex catalyzes the NADH‐dependent conversion of TPA to a non‐aromatic dicarboxylic‐diol intermediate. Decarboxylation and dehydrogenation to PCA are then carried out by a *cis*−1,2‐dihydrodiol‐TPA dehydrogenase, regenerating NADH [32, 80, 81, 82, 83, 84, 85, 86]. Recently, the crystal structures and detailed biochemical characterization of the oxygenase subunits of TPADOs from *Comamonas* sp. E6 and *Comamonas testosteronii* KF1 have been reported, providing a fundamental understanding of the reaction mechanism and substrate specificity of these enzymes [87, 88]. These works highlight that the evaluated TPADOs are highly specific towards *para*‐dicarboxylate substrates, but they are relatively slow in absolute terms (*K*_M_ of 10–70 μM, *k*_cat_/*K*_M_ of 57–75 mM^−1^ s^−1^ toward TPA) [87, 88, 89]. Nonetheless, these findings may help identify key aspects for the rational design of more efficient biocatalysts for the conversion of TPA to PCA. The second enzymatic step in TPA conversion to PCA has also been investigated to some extent, including the biochemical characterization of the *cis*−1,2‐dihydrodiol‐TPA dehydrogenase TphB from *Comamonas testoteroni* T‐2 and the crystal structure of the ortholog from *Paraburkholderia xenovorans* LB400 (formerly *Burkholderia xenovorans* LB400) [90, 91]. These studies show that a catalytic Zn^2+^ ion is essential for the binding and dehydrogenation of the *cis*‐dihydrodiol intermediate, resulting in the formation of a transient keto group, which is rapidly lost as CO_2_ to generate PCA.

Similar to TPA, IPA is first dihydroxylated by a two‐component Rieske‐type isophthalate 3,4‐dioxygenase system (IPADO), using NADH as cofactor. Likewise, the 3,4‐dihydrodiol‐IPA intermediate is decarboxylated and dehydrogenated to PCA in a second enzymatic step with the concomitant regeneration of NADH. However, contrary to TPADO, IPADO presents a single oxygenase unit. To our knowledge, the genes involved in the aerobic catabolism of IPA have only been validated in *Comamonas testosteronii* YZW‐D and *Comamonas* sp. E6 [80, 92], despite the fact that homology searches in sequence databases return hits with high sequence identity to the proteins encoded by these operons (results not shown). Furthermore, no biochemical characterization of purified enzymes has been reported. Compared to the TPA and OPA isomers, the biodegradation of IPA has been much less studied, possibly a reflection of its lower industrial relevance.

Lastly, three different pathways for OPA assimilation have been described, involving Rieske oxygenase systems that catalyze the NAD(P)H‐dependent dihydroxylation of OPA in different positions: i) two‐component phthalate 4,5‐dioxygenase, with a single oxygenase unit and its cognate reductase; ii) three‐component phthalate 3,4‐dioxygenase, with two oxygenase subunits, a ferredoxin, and a ferredoxin reductase; and iii) three‐component phthalate 2,3‐dioxygenase, also with two oxygenase subunits, a ferredoxin, and a ferredoxin reductase (hereafter 4,5–, 3,4‐, and 2,3‐OPADO, respectively). Of note, 4,5‐OPADOs are more abundant in Gram‐negative bacteria, while 3,4‐OPADOs are usually found in Gram‐positive bacteria. In both cases, the degradation pathways converge in PCA. In contrast, the 2,3‐dihydroxylation pathway leads to the formation of 2,3‐dihydroxybenzoate. So far, the 2,3‐OPADO pathway for OPA assimilation has only been described in *Pseudomonas* sp. PTH10 [93].

In particular, the 4,5‐OPADO system from *Burkholderia cepacia* DB01 has been extensively studied, and the crystal structure of the reductase component was published in 1992 [94, 95, 96, 97, 98, 99, 100, 101, 102]. However, the crystal structure of a 4,5‐OPADO oxygenase component, namely from *Comamonas* sp. KF1 was only recently solved [103]. In this work, the authors find that this enzyme is also capable of using TPA as substrate, although the reaction is at least 25 times less efficient than with OPA (*k*_cat_/*K*_M_ of 22 mM^−1^ s^−1^ for TPA vs 583 mM^−1^ s^−1^ for OPA). Interestingly, the *k*_cat_/*K*_M_ value of 4,5‐OPADO_KF1_ toward TPA is only two times lower than that of TPADO_KF1_, albeit NADH consumption is poorly coupled to TPA oxidation (5:1 mol of NADH consumed per dihydroxylated product, vs 1:1 for TPADO) [103]. This fact suggests that TPA dihydroxylation by OPADO may not be physiologically relevant in this strain, which is further supported by the fact that each phthalate degradation gene cluster encodes its own transcription factor to induce expression of the catabolic pathway when the corresponding substrate is available (Figure 6). The 4,5‐dihydrodiol‐OPA dehydrogenase from *Comamonas* sp. KF1, which dearomatizes the product of the dihydroxylation reaction, has also been crystallized and biochemically characterized [104]. This study has allowed to elucidate its reaction mechanism and explain its preferred activity towards 4,5‐dihydrodiol‐OPA over 3,4‐dihydrodiol‐OPA. Lastly, preliminary characterization of the 4,5‐dihydrodiol‐OPA decarboxylases from *Comamonas testosteroni* (formerly *Pseudomonas testosteroni*) and *Pseudomonas fluorescens* PHK has also been described in early reports [105, 106, 107]. These studies demonstrated the decarboxylation of 4,5‐dihydroxy‐OPA to PCA, and additionally found unspecific decarboxylase activity toward 4‐hydroxyphthalate in the case of the enzyme from *Comamonas testosteroni* NH1000 [105].

In contrast to the 4,5‐OPA dihydroxylation pathway, little is known about the biochemical properties of the enzymes involved in the 3,4‐OPA dihydroxylation pathway. To our knowledge, only the 3,4‐OPADO from *Rhodococcus erythropolis* S‐1 has been purified and partially characterized [108]. This enzyme was found to be NADH‐dependent and relatively active towards the OPA analogs quinolinate and cinchomeronate (2,3‐ and 3,4‐pyridine dicarboxylic acids, respectively).

### 2.4 Anaerobic Degradation Pathways

**FIGURE 7:**
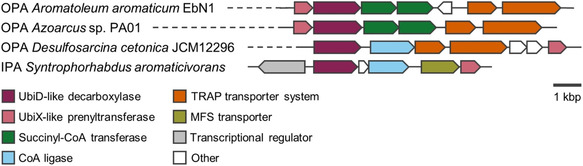
Schematic representation of the organization of representative genetic clusters for the degradation of phthalate isomers in anaerobic bacteria.

**SCHEME 2:**
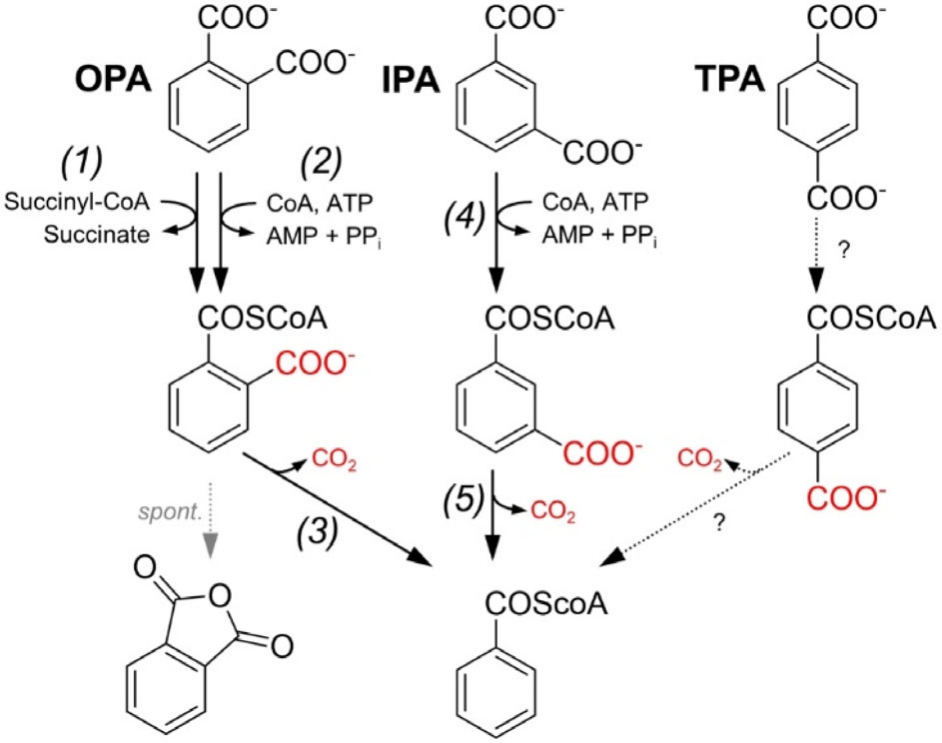
Catabolic pathways for the anaerobic assimilation of phthalates in bacteria. From left to right, OPA, IPA and hypothetical TPA pathways. Numbers in parentheses indicate the following enzymes: (*1*) succinyl‐CoA:phthalate CoA transferase; (*2*) phthalate‐CoA ligase; (3) phthaloyl‐CoA decarboxylase; (*4*) isophthalate‐CoA ligase; (*5*) isophthaloyl‐CoA decarboxylase.

In anaerobic bacteria, many aromatic compounds are converted to a benzoyl‐CoA intermediate for their assimilation. In the case of the three phthalate isomers, this involves i) activation to a CoA intermediate, and ii) decarboxylation to benzoyl‐CoA (Scheme 2). Among the three phthalate isomers, OPA degradation has been studied more extensively. In denitrifying, facultative anaerobic bacteria, such as *Azoarcus* sp. PA01, *Thauera chlorobenzoica* 3CB‐1, *Aromatoleum aromaticum* EbN1, and *Azoarcus evansii* KB470, activation of OPA to phthaloyl‐CoA is catalyzed by a succinyl‐CoA dependent CoA transferase [109, 110]. In contrast, sulfate‐reducing bacteria like the obligate anaerobe *Desulfosarcina cetonica* rely on an ATP‐dependent CoA ligase to activate OPA [111]. In particular, the succinyl‐CoA:OPA CoA transferases from *Azoarcus* sp. PA01 and *Aromatoleum aromaticum* EbN1 have been experimentally shown to be very specific toward OPA, since no activity over IPA or TPA could be detected [109, 112]. Indeed, these bacteria are unable to grow on either of these isomers. Notably, the phthaloyl‐CoA intermediate is extremely unstable and rapidly decays to phthalate anhydride by intramolecular hydrolysis (Scheme 2). This spontaneous and futile pathway is thought to be circumvented by high intracellular levels of the enzyme catalyzing the subsequent decarboxylation step: a UbiD‐like phthaloyl‐CoA decarboxylase [112, 113]. This family of enzymes utilizes a prenylated flavin adenosine mononucleotide (prFMN) as co‐factor, which is synthesized by UbiX‐like proteins [114, 115]. Genes encoding UbiD‐like phthaloyl‐CoA decarboxylase and UbiX‐like flavin prenyltransferases have been found in all known gene clusters required for the anaerobic assimilation of OPA (Figure 7).

Similar to OPA, IPA catabolism in anaerobic bacteria also proceeds via activation to isophthalyl‐CoA and decarboxylation by a UbiD‐like decarboxylase (Scheme 2). To date, only the ATP‐dependent IPA:CoA ligase from *Syntrophorhabdus aromaticivorans* has been biochemically characterized [116, 117]. Notably, this anaerobic fermenting bacterium requires a H_2_‐consuming partner in syntrophic cultures in order to degrade IPA and other aromatic compounds. As described for the anaerobic OPA degradation pathway, the IPA:CoA ligase is highly specific towards the IPA isomer. Lastly, no anaerobic TPA degradation pathways have been genetically or biochemically elucidated thus far, although anaerobic degradation of TPA is presumed to proceed through analogous coenzyme A activation and decarboxylation steps.

### 2.5 Phthalate Transport Across the Cell Membrane

As mentioned above, only monoesters and diesters of phthalates of low molecular mass are thought to be internalized by microbial cells, whereas polyesters must first be hydrolyzed in the extracellular milieu. However, little is known about their transport mechanisms. To date, only the PatDABC ABC‐transporter system from *Rhodococcus jostii* RHA1, which mediates uptake of OPA in this bacterium with a *K*_M_ of 22 μM, has been suggested to also participate in the transport of monoalkyl OPA esters, since mutants deficient in the ATP‐binding component PatB were unable to grow on OPA, monomethyl OPA, monobutyl OPA, or monohexyl OPA [48]. Nevertheless, the uptake rates for monoalkyl OPA esters in wild‐type and mutant strains were not determined, and thus the specificity of this transporter toward these substrates remains unknown.

Another possible route of entry for PAEs and other diesters and monoesters of phthalates into the cytoplasm is via passive diffusion through the cell membrane. These compounds are generally hydrophobic, with hydrophobicity increasing with alkyl chain length. Thus, they have been shown to interact closely with different components of the cell wall, including the peptidoglycan, proteins, and, in the case of Gram‐negative bacteria, outer membrane lipids. Indeed, molecular dynamics simulations of the interaction of PAEs with a lipid bilayer model have shown that these can spontaneously enter the cell membrane, with smaller PAEs like dimethyl phthalate being able to permeate and cross to the other face of the bilayer more easily [26]. This ability to spontaneously permeate the cell membrane has also been proposed for uncharged, lignin‐related aromatic model compounds, suggesting that passive membrane transport is sufficient to be physiologically relevant for sustained bacterial catabolism [118]. However, simulations have also shown that the partitioning of PAEs within the lipid bilayer could negatively affect membrane permeability and fluidity, which is in agreement with experimental observations of severe cell wall damage in bacteria grown in the presence of PAEs [27].

**FIGURE 8:**
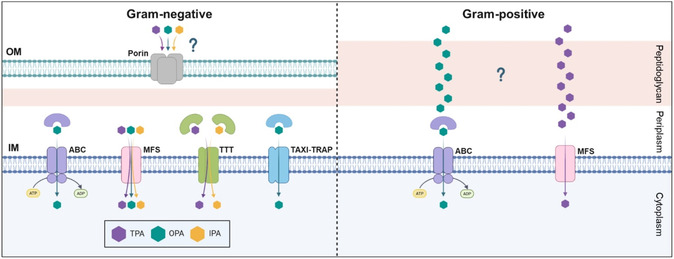
Known transport systems for the uptake of the three phthalate isomers in bacteria. Abbreviations: OM, outer membrane; IM, inner membrane. Other abbreviations are detailed in the main text.

By contrast, several transporters for the three phthalate isomers have been identified and experimentally validated. At neutral pH, the carboxylic groups of phthalates will present a negative charge, limiting their diffusion through the cell membrane. Therefore, specialized transporter systems are required. To date, four different transport systems are known to mediate the uptake of phthalates: the major facilitator superfamily (MFS), the tripartite tricarboxylate transporter system (TTT), the ABC‐transporter system, and the TRAP‐associated extracytoplasmic immunogenic‐Tripartite ATP‐independent periplasmic system (TAXI‐TRAP) (Figure 8). These transporters translocate substrates across the cytoplasmic membrane. Therefore, in the case of Gram‐negative bacteria, an additional porin or equivalent protein would be required so that substrates can cross the outer membrane [119].

MFS transporters are one of the largest families of transport proteins and are found in all kingdoms of life, using solute gradients across the membrane as driving force for the translocation of their cognate substrates [120]. MFS transporters are encoded within the genetic clusters for TPA degradation in both Gram‐negative and Gram‐positive bacteria, as well as in the gene cluster for OPA utilization in *Comamonas* sp. E6 and *Burkholderia* strains and IPA assimilation in *S. aromaticivorans* (Figures 6 and 7). Notably, in the case of *B. cepacia* DB01, the gene encoding the OPA MFS transporter presents a frame‐shift that is predicted to result in a non‐functional protein, although reversion of this frame‐shift enables OPA transport when expressed heterologously in *E. coli* [121]. This observation led to the discovery that *Burkholderia* strains encode a complementary ABC‐system for OPA uptake (see below) [122]. Similar to the catabolic genes, the MFS transporters that mediate uptake of the same substrate present high sequence similarity at the amino acid level. For example, the TPA MFS transporter from *P. xenovorans* LB400 is 49%–50% identical to the orthologs from *P. umsongensis* GO16 and *R. jostii* RHA1, whereas the latter two are 69% identical.

ABC transporters are another superfamily of proteins that are found in all taxa, mediating the ATP‐dependent import and export of molecules across the cell membrane. These transporter complexes are composed of one or two transmembrane subunits, one or two nucleotide binding subunits with ATPase activity, and, in the case of uptake systems in bacteria, a solute binding protein that specifically recognizes the substrate. ABC transporters mediating OPA uptake have been characterized in *Burkholderia* and *Rhodococcus* strains [48, 122]. Whereas the OPA ABC‐transporter from *Burkholderia* contains only one transmembrane, one nucleotide‐binding, and one solute‐binding subunits, the OPA ABC‐transporter from *Rhodococcus* contains two transmembrane subunits [48, 82]. In *Burkholderia multivorans* ATCC1616, a gene encoding an OPA porin is co‐transcribed with the genes encoding the ABC transporter. Notably, the generation of different deletion mutants showed that the MFS transporter OphD is more important than the ABC transporter for growth on OPA, although the latter has a higher affinity toward this substrate. These observations suggest that the latter is especially relevant when OPA is only available in low concentrations, whereas import through the ATP‐independent MFS transporter is favored when substrate concentrations are high [122].

The TTT system has been found to be involved in the uptake of TPA and IPA in Gram‐negative bacteria. Members of the TTT family, especially abundant in β‐proteobacteria, are composed of one periplasmic solute binding protein (SBP) and two transmembrane proteins that form the pore through which the substrate is translocated across the cytoplasmic membrane [123]. These transporters are ATP‐independent and use ion‐electrochemical gradients as driving force. The transmembrane pore proteins can interact with different SBPs, which are responsible for determining substrate specificity. Thus, the genes encoding SBPs can be localized in a different genomic locus from the ones that encode the transmembrane proteins. For example, in the case of *Comamonas* sp. E6, the genes encoding the SBP for TPA (*tphC*) and IPA (*iphC*) are found within the respective degradation clusters, whereas the genes encoding the transmembrane proteins (*tpiBA*) are located elsewhere [124]. Indeed, expression of the *tph* operon from *Comamonas* sp. E6 in a related *Comamonas testosteronii* strain, unable to natively utilize TPA, was sufficient to enable growth of the latter on this substrate, since the heterologous TphC could presumably interact with the native TpiBA transmembrane proteins from the host strain [85]. Recently, the crystal structures of TphC from *Comamonas* sp. E6 in apo and TPA‐bound conformations were solved [125].

Lastly, a TAXI‐TRAP transporter has been recently described to mediate OPA uptake in denitrifying bacteria [126]. Similar to the TTT system, TAXI‐TRAP transporters are ATP‐independent. However, unlike TTT and TRAP systems, most TAXI‐TRAP transporters comprise one SBP and a single transmembrane protein, hypothesized to be a fusion or ancestral form of the two transmembrane proteins found in the closely‐related TRAP transporters [123]. In the case of the known OPA degradation gene clusters from denitrifying bacteria, the genes encoding both components of the TAXI‐TRAP transporter are found in close proximity to the catabolic genes (Figure 7). Interestingly, orthologs possibly mediating OPA uptake in the Gram‐negative *Pseudomonas* sp. PTH10 are also found within the gene cluster for OPA utilization through the 2,3‐dihydroxylation pathway [93].

## 3 Biotechnological Opportunities for the Bioremediation, Recycling, and Valorization of Phthalates

The environmental and health‐related challenges caused by phthalate pollution have encouraged the development of new approaches for the bioremediation, recycling, and upcycling of polyesters and diesters derived from phthalates. Microbial biotechnology has received special attention as an approach with reduced environmental impact that can help develop more sustainable bioprocesses that drive a circular bioeconomy. However, these methods still present limitations. For example, slow and limited degradation efficiency, difficulty in upscaling, or high process costs. This section highlights advances and opportunities in the use of native and engineered microorganisms and their enzymes to enhance the degradation of phthalate‐related products in the environment, as well as their enzymatic recycling and microbial upcycling to high‐value‐added bioproducts. Limitations and future research directions for these emerging fields are also discussed.

### 3.1 Bioremediation Strategies

As described above, the accumulation of phthalate‐derived compounds in the environment has allowed the isolation and identification of various microorganisms capable of degrading them. Particularly, the degradation of PAEs has been documented in more than 80 bacterial strains from 36 genera, which results from several decades of research on the microbial degradation and bioremediation of PAEs. The biodegradation of PAEs in different environments has been extensively reviewed elsewhere [24, 25, 77, 127, 128, 129]. PAEs are ubiquitous pollutants that can be found in air, soil, and water bodies at concentrations that are typically below the microgram per kilogram range, although much higher concentrations (hundreds of milligrams per kilogram) can be found in sediments, digested sludge from wastewater treatment plants, or agricultural soils [130, 131, 132, 133]. The rates of accumulation and biodegradation of PAEs in the environment depend on their molecular mass and hydrophobicity. For example, high‐molecular weight DEHP (historically the most used plasticizer worldwide) is present at higher concentrations in solid matrices than in water due to its tendency to adsorb to particles, and is therefore less bioavailable than low‐molecular weight PAEs. It is also less susceptible to enzymatic hydrolysis due to steric constraints. Thus, one of the main limitations to the bioremediation of PAEs is to find microorganisms with broad tolerance, degradation capacity, and substrate diversity. Although single isolated strains with broad substrate range have been identified, especially in the case of Gram‐positive Actinobacteria, more efficient degradation of PAEs is usually achieved using microbial consortia. This is thanks to the complementation of their different metabolic capacities that can favor substrate mobilization (e.g., production of surfactants) and avoid the accumulation of degradation intermediates.

The biodegradation of PAEs in different matrices has been studied using isolates from very diverse sources, including fresh and marine water, soil, sediments, compost piles, etc. In general, removal rates that range from 40% to more than 90% are achieved within days or weeks, and is much faster in aerobic than anoxic environments [24, 25, 127, 129, 131]. PAE‐degrading organisms have also been isolated from unusual and more extreme environments, such as saline soil or mangrove sediments [134, 135, 136, 137]. However, most of these biodegradation studies have been carried out under laboratory conditions, limiting their use to controlled and closed systems. Indeed, one of the most straightforward strategies to avoid the release and dispersion of PAEs into open environments lies in developing more efficient biodegradation processes in wastewater treatment plants, e.g. with activated sludge [129, 131]. Regrettably, there are other sources of PAEs that are more difficult to contain, such as those used in fertilizers and pesticides in agricultural land. In this case, bioaugmentation (exogenous addition of live microorganisms) and biostimulation (addition of nutrients to promote growth of the indigenous microbiota) represent a promising approach [138]. An example of bioaugmentation is described by Lü et al., who constructed a synthetic bacterial consortium composed of five isolates from the corn rhizosphere for the degradation of PAEs. This consortium was able to efficiently degrade DBP and DEHP (200–500 mg/L). In addition, the synthetic consortium successfully recolonized the rhizosphere in pot experiments, showing significantly higher abundances of its genera compared to the non‐treated soil [139]. Nevertheless, the application of bioaugmentation strategies in open environments typically faces challenges such as competition between microorganisms and adaptability to changing external factors (i.e., pH, temperature, oxygen, water and micronutrient availability, etc.) [140].

In the case of PET, effective bioremediation strategies using indigenous microorganisms are highly unlikely due to the extremely slow degradation rates of this synthetic polymer under environmental conditions. As discussed above, most post‐consumer PET from textiles and beverage bottles is too crystalline to be enzymatically degraded, so the ability to significantly reduce PET pollution in open environments through bioaugmentation is questionable [22]. Indeed, any extent of biodegradation would likely be limited to MNPs in which crystallinity is reduced by abiotic weathering. Alternatively, genetically‐modified organisms (GMO) with improved PET degradation capacities could be used, but microorganisms that have been domesticated in the laboratory are usually challenged to thrive in open environments in competition with the indigenous microbiota. Another interesting approach is genetic bioaugmentation, which consists on the use of conjugative plasmids that can be transferred to bacteria that are naturally present in the target environment. Proof‐of‐concept studies have been reported for the delivery of genes encoding PET hydrolase and TPA transport and catabolism in wastewater treatment plant‐ and soil‐dwelling bacteria, respectively [141, 142]. However, the applicability of this approach in real‐world environments remains to be seen, especially considering the strict regulations on the release of GMO in many countries.

### 3.2 Enzyme Engineering for Polyester Recycling and PAE Degradation

In recent years, the chemical recycling of PET (i.e., the recovery of repolymerizable monomers) has gained significant attention as it allows obtaining recycled materials that retain the same properties as virgin PET, in contrast to mechanical recycling. Although numerous hydrolases capable of cleaving the ester bond in PET have been described, they typically show limited depolymerization yields [29]. However, intensive protein engineering and computational design efforts over the last two decades have allowed obtaining improved enzymes that are suitable for the enzymatic recycling of PET at commercial scale [30].

As mentioned, the main limitation to the enzymatic depolymerization of PET is its crystallinity, which limits the access of the enzyme to the polymer chain. To an extent, chain mobility can be improved by performing the depolymerization reaction near the glass transition temperature of PET (˜70°C). Thus, thermostable enzymes are needed. A major breakthrough in the field was the demonstration of post‐consumer PET enzymatic recycling at pilot scale (150‐liter reactor) using an engineered variant of the thermophilic leaf‐branch compost cutinase (LCC) [143]. Through rational design and saturation mutagenesis of selected residues of the active site of LCC, the LCC^ICCG^ variant improved melting temperature (*T*_m_) by 9.3°C (*T*_m_ = 94°C) and achieved 90% conversion of amorphized and micronized PET in less than 10 h at 72°C, with a productivity of 16.7 g TPA/L/h. This process has served as basis for the implementation of enzymatic recycling of post‐consumer PET at demonstration scale.

Impressive improvements to the activity and thermostability of the mesophilic *Is*PETase have also been obtained through rational and computational design or directed evolution. In an early work, Son et al. describe the rational design of ThermoPETase, which accommodates three amino acid replacements that improve the *T*_m_ by almost 9°C with respect to wild‐type *Is*PETase (*T*_m_ = 48.8°C) [144]. Cui et al. used the GRAPE (Greedy Accumulated Strategy for Protein Engineering) computational strategy to obtain DuraPETase, which exhibited an apparent *T*_m_ increase of 31°C (*T*_m_ = 77°C) [145]. This variant also improved 300‐fold the degradation of semi‐crystalline PET films (30% crystallinity) at 37°C, although the overall conversion was still low (15% after 10 days). Along these lines, Lu et al. used a structure‐based machine learning algorithm to identify stabilizing mutations using the ThermoPETase variant as scaffold, obtaining FAST‐PETase [146]. FAST‐PETase outperformed other engineered *Is*PETase and LCC variants at 30°C–50°C using an amorphous PET substrate. Additionally, the authors demonstrated the enzymatic recycling of a post‐consumer PET container at 50°C using FAST‐PETase, although the process lasted 6 days and required the serial addition of enzyme every 24 h. However, the highest increase in thermostability was achieved by directed evolution. Also using ThermoPETase as scaffold, Bell and colleagues employed saturation mutagenesis over 106 of the 264 residues of *Is*PETase, applying increasing reaction temperature and time as selection pressure [147]. The resulting HotPETase variant, which accumulates 21 amino acid substitutions with respect to wild‐type *Is*PETase, presented a *T*_m_ of 82.5°C. Depolymerization of semicrystalline PET powder at 60°C was faster using HotPETase than LCC^ICCG^, although final depolymerization yields after 48 h were comparable. Of note, MHET was the major depolymerization product for most tested conditions when using HotPETase.

Despite all the improvements made to the stability of *Is*PETase, a systematic comparison of four engineered enzymes (LCC^ICCG^, FAST‐PETase, HotPETase, and PES‐H1^L92F/Q94Y^) under standardized conditions revealed that variants derived from naturally thermostable PET hydrolases achieved higher depolymerization yields, even when each enzyme was assayed at its optimum temperature [148]. This study highlights the need to standardize assays to compare the performance of different enzyme variants for PET recycling, taking into consideration the intrinsic properties of the substrates that will realistically be used in a commercial process [30]. In this sense, it is worth mentioning some recent works describing the computation‐guided engineering of newly isolated thermophilic PET hydrolases that may also be well‐suited for industrial PET enzymatic recycling. Namely, metagenome‐derived PES‐H1 and BhrPETase [149, 150]. Nevertheless, the use of mesophilic PET hydrolases that are more efficient at mild temperatures could still be relevant in other contexts, such as the elimination of PET MFs and MNPs during wastewater treatment. In this case, stability over long periods of time would be desirable, as well as enzyme immobilization to minimize the loss of biocatalyst.

Indeed, several studies have addressed the immobilization of PET hydrolases on both abiotic and biological scaffolds. For instance, immobilization of *Is*PETase or derived FAST‐PETase variant on magnetic nanoparticles has been shown to increase enzymatic stability, improving depolymerization yields compared to free enzymes in solution and further allowing the simple recovery of active enzyme over multiple cycles [151, 152, 153]. Likewise, immobilization of PETase on mesoporous silica nanocarriers improved the degradation of PET microfibers under simulated wastewater conditions [154]. Improved hydrolytic activity is thought to result not only from enhanced stability, but also from increased local concentration of enzyme at the PET interface. In agreement with this hypothesis, cell surface display of PETase in yeasts has also been shown to improve depolymerization of highly crystalline PET [155, 156, 157]. This improvement is dramatically enhanced (>300‐fold over free enzyme) with the codisplay of hydrophobins, which favor the attachment of yeast cells to the hydrophobic surface of PET [156].

By comparison, only a small number of PAE hydrolases have been characterized in detail and, accordingly, there are very few available studies in which PAE hydrolases have been engineered for improved performance. In 2021, Qiu et al. generated a random variant library of the diesterase/monoesterase EstJ6 with two rounds of error‐prone PCR, which was screened for improved DBP hydrolysis [158]. The best mutant showed 2.8‐fold increase in enzymatic activity and improved thermostability compared to wild type. Interestingly, the three selected amino acid substitutions (A67V, T91M, and V249I) are located in the α/β hydrolase core but far away from the active site. Subsequent molecular dynamic simulations predicted that these substitutions may affect the flexibility of the cap domain, suggesting an effect on substrate binding [159].

In another work, 5000 variants of the diesterase GoEst15 were screened for improved activity toward DBP with a fluorescent biosensor capable of detecting OPA [160]. Monoesterase GoEstM1 was co‐expressed in order to hydrolyze the monoester. In this case, 49 residues surrounding the active site were selected for saturation mutagenesis. The selected variant GoEst15‐V3 showed twofold higher specific activity towards DBP with respect to wild type. Furthermore, it achieved 96.5% hydrolysis of 5 mM DBP in 60 min, while the wild‐type enzyme degraded only 55%.

In view of the great milestones that have been accomplished in the engineering of more robust and active PET hydrolases, it is likely that similar progress can be made for PAE hydrolases for the degradation of toxic plasticizers. Future research directions should focus on understanding the determinants of catalytic efficiency in PAE hydrolases (e.g., substrate binding, steric constraints, diesterase versus monoesterase activity), improving activity toward high‐molecular‐weight PAEs such as DEHP, improving enzyme stability for prolonged activity in PAE degradation treatments, and devising strategies for the immobilization or delivery of active enzymes to treat polluted environments.

### 3.3 Whole‐Cell Engineering for Phthalate Valorization

New metabolic engineering and synthetic biology approaches have allowed the development of biofactories from microbial cells with the ability to convert environmental pollutants such as phthalates into high value‐added products. Their conversion into valuable compounds could not only mitigate their environmental impact, but also generate an economic revenue to promote a circular economy [161, 162]. Similar to enzymatic recycling processes, current advances in the bio‐upcycling of phthalates have largely focused on TPA as a monomer of PET (Table 2).

**TABLE 2:** Genetically engineered bacteria for the upcycling of PET and derived compounds. Maximum production titers and molar yields are those reported in the original works.

Host	Relevant genetic modifications	Substrate	Target compounds	Maximum Titer, T/Molar yield, Y	Reference
*E. coli* (Resting cell)	TPA catabolic genes from *Comamonas* sp. E6; heterologous genes for conversion of PCA; synthetic consortium	Chemical PET hydrolysate (TPA + EG)	Gallic acid, pyrogallol, muconic acid, vanillic acid	Y: 92.5%, 32.7%, 85.4%, 41.6%	[163]
*E. coli* (Resting cell)	TPA catabolic genes from *Comamonas* sp. E6; heterologous genes for vanillin production from PCA	TPA; Enzymatic PET hydrolysate (TPA + EG)	Vanillin	T: 119 mg/L; Y: 79%	[164]
*E. coli* (Resting cell)	TPA catabolic genes from *Comamonas* sp. E6; heterologous genes for vanillin production from PCA; deletion of genes encoding cell membrane proteins	TPA; Enzymatic PET hydrolysate	Vanillin	T: 258 mg/L; Y: 71%	[165]
*E. coli* (Resting cell)	TPA catabolic genes from *Comamonas* sp. E6; heterologous genes for adipic acid production from PCA; alginate immobilization	TPA; Chemical PET hydrolysate	Adipic acid	T: 115 mg/L; Y: 79%	[166]
*E. coli* (Resting cell)	Deletion of genes for PABA utilization; heterologous genes for paracetamol production from PABA; single‐strain or synthetic consortium	4‐carboxylate‐O‐ pivaloyl benzhydroxamic acid from chemical PET hydrolysate	Paracetamol	Y: 92%	[167]
*P. umsongensis* GO16	Laboratory‐evolved strain for growth on EG; heterologous genes for HAA production	Enzymatic PET hydrolysate (TPA + EG)	PHA, HAA	T: 14 mg/L, 35 mg/L	[168]
*P. putida* KT2440	PETase and MHETase genes from *I. sakaiensis*; TPA catabolic genes from *Comamonas* sp. E6; TPA transport gene from *R. jostii* RHA1; deletion of repressor and overexpression of native genes for growth on EG	BHET; Chemical PET hydrolysate (BHET); Glucose co‐feeding	β‐ketoadipic acid	T: 15.1 g/L; Y: 76%	[169]
*P. putida* KT2440	TPA catabolic and transport genes from *R. jostii* RHA1; deletion of repressor and overexpression of native genes for growth on EG; heterologous genes for muconic acid production from PCA; synthetic consortium	Chemical PET hydrolysate (TPA + EG)	PHA, muconic acid	T: 637 mg/L, 4.73 g/L	[170]
*P. putida* KT2440	TPA catabolic and transport genes from *P. umsongensis* GO16; deletion of repressor for growth on EG; deletion/overexpression of genes for growth‐coupled PHA production	Synthetic mixture of TPA and EG; Enzymatic PET hydrolysate (TPA + EG)	PHA	T: 12.38% g/g_CDW_	[171]
*P. putida* KT2440	TPA catabolic and transport genes from *P. umsongensis* GO16; deletion of repressor for growth on EG; heterologous genes for cyanophycin, HAA, and rhamnolipid production	Synthetic mixture of TPA and EG; Enzymatic PET hydrolysate (TPA + EG)	Cyanophycin, HAA, rhamnolipids	T: 1.4 g/L, 245 mg/L, 385 mg/L	[172]
*P. putida* KT2440	TPA catabolic genes from *Comamonas* sp. E6; TPA transport gene from *Pseudomonas mandelli* JR1; heterologous genes for protein production	TPA	Recombinant therapeutic proteins	n.d.	[173]
*R. jostii* PET	Overexpression of genes for lycopene and lipids production	Chemical PET hydrolysate (TPA + EG)	Lycopene, lipids, succinate.	T: 22.6 mg/L, 2.08 g/L, 2.15 g/L	[174, 175]

Abbreviations: CDW: cell dry weight; EG: ethylene glycol; HAA: hydroxyalkanoyloxy‐alkanoate; PABA: *p*‐aminobenzoic acid; PCA, protocatechuic acid; PHA, polyhydroxyalkanoate;

*Note:* n.d.: not determined. Other abbreviations are described in the main text.

**FIGURE 9:**
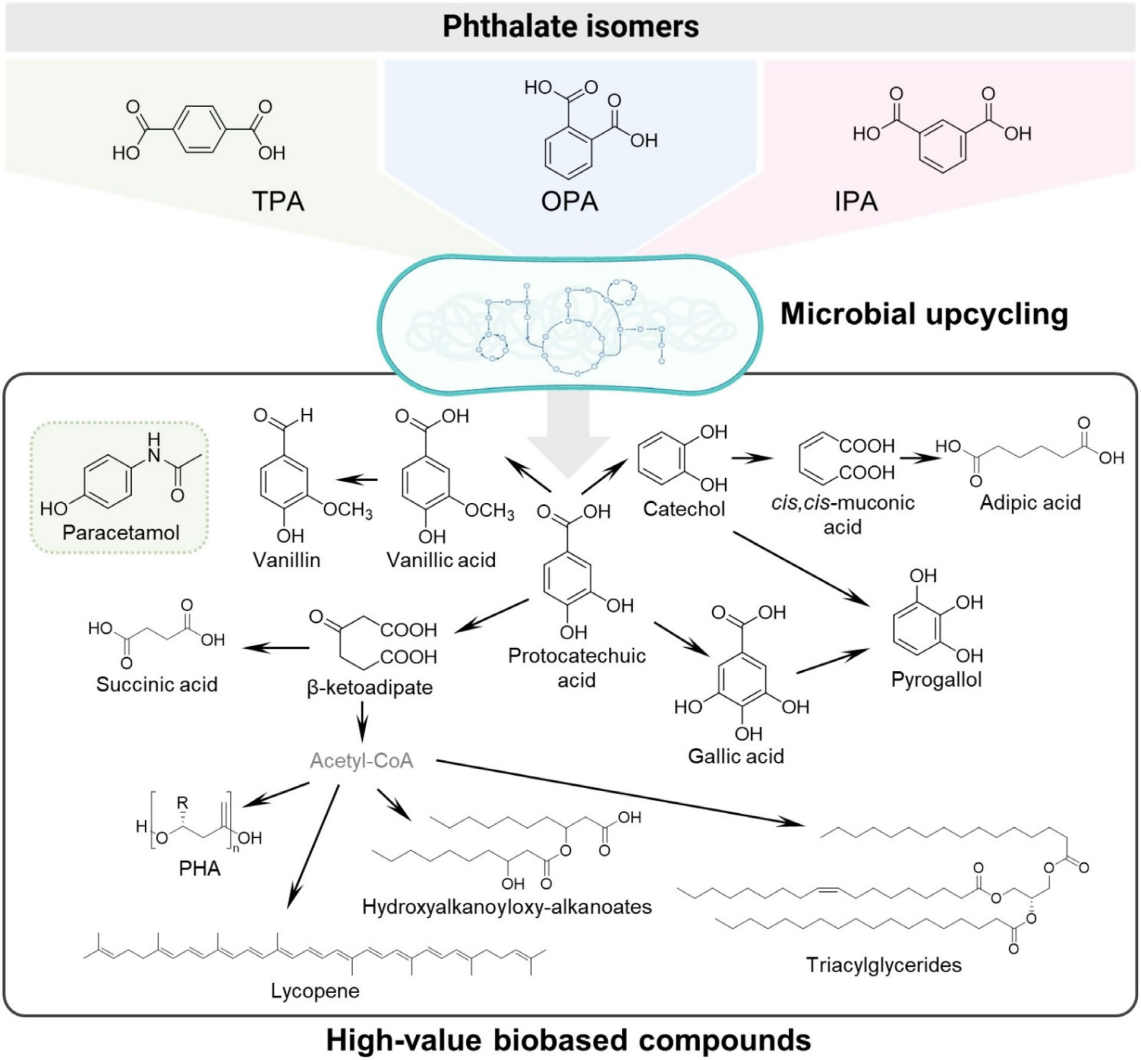
Value‐added products obtained from the biological valorization of phthalates (see Table 2). Aerobic catabolic pathways for phthalates converge into protocatechuic acid, which can be converted into other aromatic molecules or intermediates derived from aromatic ring cleavage. If fully catabolized to acetyl‐CoA, this can serve as starting point for the synthesis of lipids and terpenes. In the case of TPA, it can be chemically converted into an acyl hydroxamate intermediate for the biological production of paracetamol.

In 2008, Kenny et al. first reported the ability of several *Pseudomonas* isolates to upcycle PET‐derived TPA into polyhydroxyalkanoate (PHA), a natural high‐value biopolymer that can be used as a biodegradable replacement for conventional plastics [176]. Since then, many efforts have been dedicated to engineering new microbial hosts for the upcycling of TPA and expanding the portfolio of bio‐derived products that can be generated from this substrate (Table 2 and Figure 9). Early works described the development of multi‐enzyme cascade systems in resting cells of *E. coli* to convert TPA to different value‐added compounds, such as vanillic acid, gallic acid, pyrogallol, and catechol [163]. Similar strategies have been used to produce vanillin, which is widely used as flavoring in the food industry, and adipic acid, a platform chemical used as a precursor for nylon‐6,6, among other uses [164, 165, 166]. Notably, no heterologous TPA transporters were introduced in these works, such that cell membranes had to be permeabilized chemically or through the deletion of membrane proteins to improve TPA uptake. More recently, a hybrid biological‐catalytic approach, also based on *E. coli* resting cells, was developed to produce paracetamol from a TPA‐derived acyl hydroxamate [167].

An alternative to the use of resting cells as catalysts for the bioconversion of TPA has been to couple growth on TPA to the production of the target molecule. In this way, *Pseudomonas* strains such as *P. putida* KT2440 and *P. umsongensis* GO16 have been engineered to produce high‐value compounds including PHA, 3‐(3‐hydroxyalkanoyloxy) alkanoates, recombinant therapeutic proteins, cyanophycin, rhamnolipids, and β‐ketoadipic acid [168, 169, 170, 171, 172, 173]. In addition, Gram‐positive bacteria, namely from the *Rhodococcus* genus, have also been engineered for the valorization of PET‐derived TPA. Recent works describe the implementation of genetic engineering tools in the newly isolated *Rhodococcus jostii* PET strain, which naturally grows on TPA, to produce lycopene, lipids, and succinate from PET waste residues [174, 175]. Overall, these works highlight the enormous potential of microbial engineering and synthetic biology to harness the metabolic versatility of bacteria to produce a variety of high‐value chemicals, which can contribute to the upcycling of post‐consumer PET waste.

It is worth mentioning that state‐of‐the‐art approaches that complement conventional metabolic engineering have helped improve the yields of PET bioconversion. For example, Bao et al. developed a synthetic consortium of two *P. putida* strains, each specialized in the conversion of TPA or EG [170]. This division‐of‐labor strategy allowed producing almost twofold PHA and 2.8‐fold muconic acid with respect to the control strain capable of co‐consuming TPA and EG, achieving final titers of 637 mg/L and 4.73 g/L from ˜50 mM PET hydrolysate (11.4 g/L), respectively. On the other hand, in silico tools based on genome‐scale metabolic models may also aid in the design of more efficient bioconversion strains by predicting the optimal distribution of metabolic fluxes. Manoli et al. employed a model‐driven strategy to couple PHA production to growth on an enzymatic PET hydrolysate in *P. putida* [171]. Typically, PHA are only produced as carbon storage compounds under nutritional stress when the cells are not actively growing, which results in longer cultivation times. In silico predictions in combination with synthetic biology tools to optimize gene expression allowed the authors to redirect carbon flux toward PHA biosynthesis during the growth phase, achieving 11.06% PHA production over cell dry weight compared to 7.14% produced by the non‐optimized strain. Lastly, adaptive laboratory evolution (ALE) may aid in the agnostic multifactorial optimization of engineered strains to improve their performance. ALE of a previously engineered *P. putida* KT2440 strain improved growth rate on mixed TPA and EG 3.5‐fold. Whole‐genome resequencing and reverse engineering confirmed the contribution to increased growth rates on TPA of the deletion of genes encoding three global regulators (GacA, GacS, and TurA) and the overexpression from duplicated copies of the heterologous *tpaK* transporter and *tphAB*_*II*_ catabolic genes [177].

Remarkably, the engineering of heterologous TPA catabolic pathways in non‐native TPA degraders has highlighted transport as a limiting step. In an early work, Sasoh et al. demonstrated that co‐expression of *tphC*—encoding the SBP of the TPA TTT‐transporter from *Comamonas* sp. E6—was required to enable growth of an engineered *Comamonas testosteroni* IAM 1152 strain expressing the catabolic genes from the same donor [85]. By contrast, this phenotype was not replicated in *P. putida*, which required the additional introduction of the *tpiBA* genes encoding the transmembrane components of the TTT‐transporter to uptake TPA. However, this strain was still unable to grow on TPA as sole carbon source [124]. Efficient transport and growth on TPA for a non‐related heterologous host was first achieved in *Acinetobacter baylyi* ADP1. Using the Evolution by Amplification and Synthetic Biology method (EASy), Pardo et al. evolved *A. baylyi* strains expressing genes for TPA transport and catabolism from *Comamonas* sp. E6 for growth on TPA. Subsequent experiments revealed that the heterologous TTT transporter was not functional in the host strain, and that mutations selected in a native MFS transporter (namely MucK) resulted in more efficient TPA uptake [178]. Analogous findings have been made in *P. putida* KT2440. Werner et al. observed that only the combination of catabolic genes from *Comamonas* sp. E6 and the MFS transporter gene *tpaK* from *R. jostii* RHA1 allowed growth on TPA [169]. Brandenberg et al. also identified a point mutation in the native MFS transporter MhpT that enabled TPA uptake by a *P. putida* KT2440 strain expressing the catabolic genes from *Comamonas* sp. E6 [179]. These findings highlight the importance of identifying and incorporating specialized transporters that are compatible with the host strain in order to obtain efficient microbial cells for bioconversion applications.

On the other hand, the upcycling of IPA and OPA has not been sufficiently explored to date. However, their potential is promising due to the convergence of their catabolic pathways with those of TPA. The three isomers can be metabolized into common intermediates, suggesting that, using engineering strategies similar to those developed for TPA, it may be possible to produce the same value‐added compounds from the three isomers (e.g., biopolymers, surfactants, or bioplastic precursors). An example of this possibility was reported by Sanz et al., where they demonstrated that the heterologous co‐expression of OPA catabolism and TAXI‐TRAP transport genes from *A. aromaticum* Ebn1 in *Cupriavidus necator* H16 strain enabled production of PHA from OPA [126]. This represents an opportunity for the simultaneous use of phthalate mixtures derived from plastic waste, promoting a more circular and sustainable economy. However, to achieve a successful valorization of phthalates, it is essential to continue multidisciplinary efforts aimed at improving the performance of microbial strains and scaling up these biotechnological processes [161, 162].

### 3.4 Phthalate Biosensors

**FIGURE 10:**
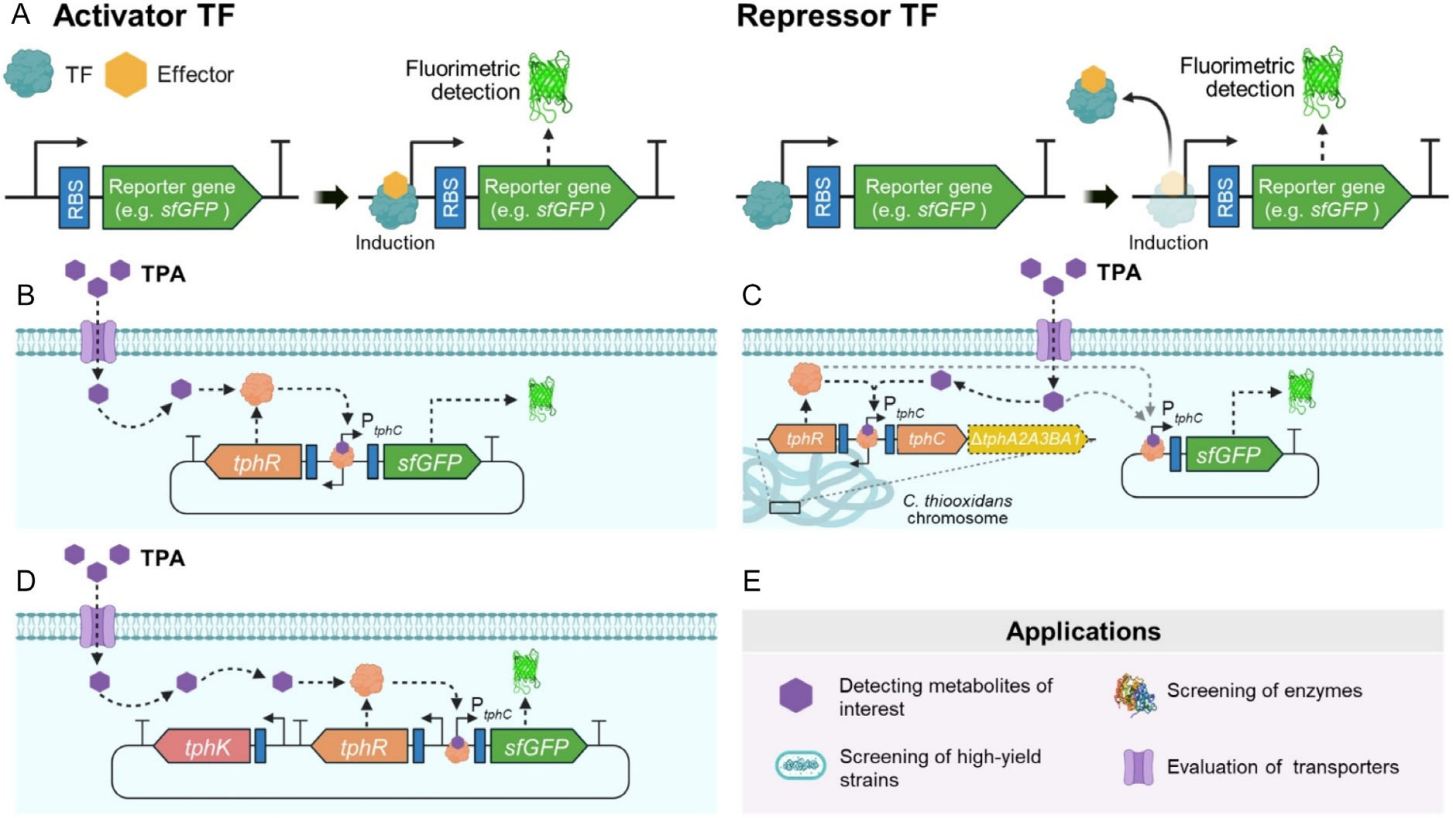
Biosensors for the detection of phthalates in bacteria. (A) General overview of the mechanism of transcription factors (TF) acting as activators or repressors. (B–D) Schematic representation of biosensors developed by (B) Pardo et al., 2020 [178]; (C) Dierkes et al., 2023 [183]; and (D) Alvarez Gonzalez et al., 2024 [184]. (E) Applications of biosensors in the context of microbial degradation, recycling and upcycling of phthalates. Abbreviations: TF: transcription factor; RBS: Ribosome binding site.

Biosensors are useful genetic tools that can be used for the detection of compounds in different environments, the in vivo quantification of metabolites, the screening of enzymes, and the evaluation of cell transport [180, 181, 182]. These systems have proven to be reliable and specific in identifying a large number of molecules of biotechnological interest. Oftentimes, genetically encoded biosensors are engineered by coupling the expression of a reporter gene (e.g., a fluorescent protein) to the regulation by an allosteric transcription factor (TF) that recognizes the molecule of interest (Figure 10). In recent years, various TF‐based biosensors have been developed to detect phthalates in vivo, especially for TPA.

The first reported TPA biosensor was developed by Pardo et al*.* for *A. bayly*i ADP1 (Figure 10B). It consisted of a plasmid harboring the TphR TF and the bidirectional *tphR‐tphC* regulatory region from *Comamonas testosteroni* that regulated the expression of a reporter gene encoding the superfolder green fluorescent protein (sfGFP) [178]. TphR acts as an activator, binding an operator region upstream of the *tphC* promoter and favoring recruitment of the RNA polymerase complex. It also represses its own expression [185]. In this work, the directed evolution through random mutagenesis of different regions of the *tphC* promoter coupled to fluorescence‐activated cell sorting improved the sensitivity and dynamic range of the biosensor, which could detect 0.1–10 mM TPA in wild‐type *A. baylyi* with a sevenfold range of fluorescence response. This improved biosensor allowed them to evaluate TPA transport in vivo in different transporter mutants obtained by ALE on TPA, which further improved sensitivity to 10 µM TPA.

Other TPA biosensors have been used for the semi‐quantitative analysis of PET hydrolase activity, using sensing cells to measure released TPA from enzymatic PET hydrolysates. Dierkes et al. reported a whole‐cell TPA biosensor using *Comamonas thiooxidans* S23 [183]. This strain is a natural TPA degrader that can uptake TPA through its native TTT system. In this case, the *tphC* promoter was cloned upstream of the sfGFP coding sequence in a plasmid, whereas *tphR* was expressed from the chromosome of *C. thiooxidans* S23 (Figure 10C). The biosensor was responsive to at least 10 μM TPA, with a maximum increase in fluorescence response of 25‐fold at concentrations above 50 μM TPA. Sensitivity was improved 10,000‐fold by deleting the *tph* catabolic genes, which allowed detection of 1 nM TPA, although the dynamic range was reduced to approximately tenfold.

More recently, Alvarez‐Gonzalez et al. reported the design of fully modularized TPA biosensors in *P. putida* KT2440 [184]. In particular, they characterized five TphR orthologs from different species. To improve modularity, they decoupled the expression of the regulation module by placing *tphR* under a synthetic constitutive promoter. Furthermore, they included a *tphK* gene encoding a TPA MFS transporter in the genetic circuit to enable the uptake of the inducer in *P. putida* KT2440 (Figure 10D). This design allowed them to modulate the optimal expression of each component (transporter, transcription factor, and reporter) by screening libraries of promoter sequences and ribosome binding sites in a design‐of‐experiments approach. The best TPA biosensor demonstrated a sensitivity below the micromolar range and a dynamic range of 13.2‐fold. Furthermore, the authors demonstrated the applicability of two biosensor versions with different operation ranges to evaluate the activity of PET hydrolases: one for a YES/NO screening of TPA release and another to detect differences in activity.

In an alternative approach, a biosensor based on LuxAB luciferase from *Photorhabdus luminescens* has also been used to establish a reporter system in *E. coli* for the detection of TPA in PET hydrolysates [186]. This biosensor is not based on a transcription factor, but rather on a carboxylic acid reductase that converts TPA to an aldehyde that is substrate for LuxAB, generating a bioluminescent response. In this case, TPA uptake by the cell was facilitated with the deletion of the gene encoding the Lpp membrane protein, which increases membrane permeability. However, unlike fluorescent proteins, the luminescent signal generated by LuxAB is transient and depends to a great extent on the metabolic state of the cell, which can lead to readout variability.

Another work by Li et al. focused on the directed evolution of a TF that does not natively bind phthalates for the detection of these compounds [160]. The promiscuous XylS from *Pseudomonas putida* was engineered by several rounds of random mutagenesis to bind OPA and TPA, with the concomitant activation of the expression of sfGFP from the cognate Pm promoter. The evolved biosensor variant had a sensitivity of 10 µM toward both phthalate isomers in *E. coli*, with a dynamic range of 2.2‐ and 3.0‐fold for OPA and TPA, respectively. Furthermore, the authors demonstrated the use of this biosensor as a screening method for the directed evolution of PAE hydrolases expressed in the same sensing strain. However, the low substrate specificity of this biosensor could be a disadvantage for certain applications where a mixture of aromatic compounds is expected.

Although most of the reported works have focused on TPA, there is potential to develop highly specific biosensors for each phthalate isomer using approaches analogous to the ones described above. Indeed, the known catabolic clusters for the assimilation of TPA, IPA, and OPA encode their respective TFs that are likely to discriminate between the different isomers. In particular, the regulation of the IPA catabolic operon by the transcriptional regulator IphR in *Comamonas* sp. E6, which acts as a repressor that specifically responds to IPA, has been characterized in detail [187]. Additionally, the diversity of known OPA degradation gene clusters offers a large resource of genetic elements to design optimal biosensor circuits for the detection of this compound. However, the design and functionality of these systems will need to consider the limitations imposed by the transport of phthalates into the cell interior, which will influence both the sensitivity and response of the biosensors. The importance of this consideration is highlighted in a recent work where the evaluation of different transporters in *E. coli* greatly affected the performance of a TphR‐based biosensor for the detection of TPA [188]. In particular, the authors compared four different MFS transporters (the TTT system from *Comamonas* sp. E6 was also found to not be functional in *E. coli*): two native TPA transporters from *P. umsongensis* GO16 and *R. jostii* RHA1 (TphK and TpaK, respectively) and two variants of *A. baylyi* ADP1 and *P. putida* KT2440 transporters that had been spontaneously selected during evolution for growth on TPA (MucK and MhpT, respectively). Interestingly, the laboratory‐evolved MucK variant from *A. baylyi* ADP1 rendered the best sensitivity and dynamic range using the same biosensor circuit, suggesting that wild‐type transporters naturally evolved to uptake a certain compound may not be the most appropriate to develop whole‐cell biosensors for certain applications.

Collectively, genetically‐encoded biosensors have great potential for screening and optimizing new hydrolases with high efficiency for the degradation of PET, PAEs, and other phthalate‐based esters. They could also serve to develop cheap devices for the detection of these compounds in the environment. However, if TPA or OPA whole‐cell biosensors were to be used to this end, co‐expression of hydrolytic enzymes to release free‐monomers would be required. The direct detection of PET or toxic PAEs using TF‐based biosensors would be limited, due to the large size of the PET polymer chain and the structural diversity of the many PAEs that are used for different applications. Although in the particular case of PAEs, the development of specific biosensors could significantly improve their usefulness in the detection and biodegradation of these pollutants in the environment [189]. Lastly, a detailed understanding of these genetic circuits could also help design optimized strains for the valorization of phthalates in the context of upcycling plastic waste, by fine‐tuning gene expression and reducing the metabolic burden in engineered microbial cells.

## 4 Summary and Outlook

Despite significant progress in understanding phthalate biodegradation, several knowledge gaps and techno‐economic hurdles remain that limit its application in the bioremediation, recycling, and upcycling of plastic waste. With regards to enzymes for the hydrolysis of phthalate esters, impressive advances have been made in the last 20 years in developing technologies and robust catalysts for the enzymatic recycling of PET, which has allowed implementing these processes at the demonstration scale. In comparison, fundamental knowledge of the reaction mechanisms of PAE hydrolases is still lagging, translating into limited engineering efforts to improve the stability and efficiency of these enzymes. Using state‐of‐the‐art technologies, such as computation‐assisted protein design and directed evolution, could deliver application‐relevant biocatalysts for the bioremediation of toxic PAEs in open environments or for their controlled removal in industrial settings, such as waste water treatment plants or new recycling facilities. Indeed, the high additive content in certain plastics, such as PVC—the majority of which are PAEs—limits their recyclability by mechanical methods. Furthermore, the presence of “legacy additives” (substances previously used as additives that are now regulated as substances of very high concern) requires that these be safely removed from the materials before they can re‐enter the value chain. When this is not possible, the material must be permanently destroyed [190]. Alternative recycling technologies, such as solvent‐based dissolution‐precipitation methods, are a promising approach to favor the circularity of these types of plastics, and coupling PAE extraction to enzymatic or microbial degradation could be a solution to their safe removal [191, 192]. Furthermore, the upcycling of PAEs to value‐added products could improve the economic feasibility of these processes, as discussed below.

In any case, robust and highly active enzymes with a broad substrate range will be required for these applications. In this context, high‐throughput screening methods are extremely powerful tools to evaluate the activity of large protein variant libraries. Biosensors analogous to the ones developed for the detection of TPA and the evaluation of PET hydrolases can be developed to detect OPA released from PAE hydrolysis. This would allow screening PAE hydrolases capable of degrading a wide variety of PAEs from mutant libraries or metagenomic sources, or enzymes with improved diesterase/monoesterase activity. However, the application of OPA biosensors for the detection of PAEs in contaminated waters or soils would not be straightforward, since a first hydrolytic step would be required. Furthermore, the use of whole‐cell biosensors would present additional limitations due to transport constraints for PAEs. These limitations could be circumvented by developing biosensors that specifically bind PAEs, secreting and anchoring PAE hydrolases to the cell wall, or using cell‐free extracts. However, to date, there are no known transcription factors that specifically respond to PAEs, and the structural diversity of PAEs used in different applications can complicate the design of effective biosensors. Thus, efforts dedicated to the development of PAE‐specific biosensors should initially focus on those that are considered substances of very high concern. These biosensors could be obtained either by engineering transcription factors that bind structurally related molecules or by using alternative genetically encoded biosensors such as riboswitches. Riboswitches are structural regulatory elements typically found in the 5^′^ leader sequence of bacterial mRNA that can bind different molecules with high affinity and specificity, modulating the transcription or translation of the downstream coding sequence. There are numerous examples of de novo riboswitch design for the detection of a wide variety of effectors, including ions, metabolites, or proteins [193].

As for the assimilation of phthalates and their conversion into value‐added products, numerous catabolic pathways have been described and engineered in native or heterologous hosts. The detailed biochemical characterization of these pathways and their regulation at different levels (transcriptional, translational, and post‐translational) could aid in the design of more efficient microbial factories for the upcycling of plastic waste‐derived phthalates. Furthermore, synthetic biology and the combination of organic chemistry with cellular transformations could expand the array of molecules that can be accessed through new‐to‐nature reactions, as exemplified in the recent study by Johnson et al., where post‐consumer PET is converted to paracetamol [167]. These approaches are an exciting avenue of research that is rapidly expanding, and they have the potential to improve the economic viability of alternative recycling technologies such as chemical or dissolution‐based recycling. At present, these technologies are considered a solution to deal with post‐consumer plastic waste that is not suitable for mechanical recycling, but their lower technology readiness level and high process costs have limited their widespread implementation. However, microbial processes in themselves face numerous challenges during upscaling, such as consistent strain performance, adequate mixing of nutrients, product recovery, competitive titers, rates, and yields to ensure economic feasibility, etc [194, 195]. Overcoming these hurdles requires multidisciplinary approaches and strategic optimization of each process step to accelerate the transfer of these technologies from academy to industry.

Lastly, in situ biodegradation is a potential economically feasible and environmentally friendly strategy to eliminate phthalate‐derived pollutants from the environment, especially when their recovery for ex situ treatment is not viable [138]. However, there are limitations imposed by both material properties and microbial physiology to the bioremediation of phthalate‐derived compounds in uncontrolled environments. On one hand, PET is extremely inert to enzymatic attack under ambient conditions, and the bioavailability of PAEs is limited due to their tendency to adsorb to particles. On the other, transport constraints, enzymatic and metabolic versatility, and cofactor and nutrient availability are crucial factors that affect biodegradation, as well as changing environmental conditions and competition with the indigenous microbiota. Engineering strains or synthetic consortia using synthetic biology tools could greatly improve the effectiveness of bioremediation, but the application of genetically‐modified microorganisms will require thorough risk assessment and the implementation of appropriate regulations and containment strategies to prevent unintended ecological impacts [196, 197].

In summary, the mitigation of the environmental pollution and health concerns caused by plastic‐related phthalate products using microorganisms and their enzymes will require a multidisciplinary approach that integrates advances in biotechnology, microbial ecology, environmental engineering, and health risk assessment. However, ambitious policy changes will be needed as well. Since the pollution caused by plastics is poorly reversible, it is fundamental to prevent uncontrolled leaking into the environment and ensure that plastics and their additives are safe by design. A continuous monitoring and updating of their environmental and health risks will be needed as new alternatives enter the market. Only the integration of these strategies could accelerate the elimination of these contaminants and contribute to the transition towards more sustainable management of plastic waste and its additives.

## Author Contributions

**Marco A.** **Pereyra‐Camacho**: writing – original draft: equal; writing – review and editing: equal. **Isabel Pardo**: writing – original draft: lead; writing – review and editing: lead.

## Funding

This study was supported by Consejo Superior de Investigaciones Científicas (Grant 20210510, PIE202120E064), Fundación Reina Sofía (ES) (Grant 20210510), MICIU/AEI/10.13039/501100011033 and the European Union NextGenerationEU/PRTR (Grant TED2021‐180350A‐I00), Comunidad de Madrid (Grant 2022‐T1/BIO‐23939).

## Conflicts of Interest

The authors declare no conflicts of interest.
